# Genome-wide identification of growth-regulating factors in moso bamboo (*Phyllostachys edulis*): in silico and experimental analyses

**DOI:** 10.7717/peerj.7510

**Published:** 2019-09-12

**Authors:** Yanan Shi, Huanlong Liu, Yameng Gao, Yujiao Wang, Min Wu, Yan Xiang

**Affiliations:** 1 Laboratory of Modern Biotechnology, School of Forestry and Landscape Architecture, Anhui Agricultural University, Hefei, China; 2 National Engineering Laboratory of Crop Stress Resistance Breeding, School of Life Sciences, Anhui Agricultural University, Hefei, China

**Keywords:** Moso bamboo, GRFs, Transcription activity, Yeast two-hybridization assay, BiFC

## Abstract

Growth-regulating factor (GRF), a small plant-specific transcription factor (TF) family, is extensively involved in the regulation of growth and developmental processes. However, the GRF family has not been comprehensively studied in moso bamboo (*Phyllostachys edulis*), a typical non-timber forest member. Here, 18 *GRF* genes were identified and characterized from the moso bamboo genome, and they clustered into three subfamilies (A, B and C). *PeGRF* genes were analyzed to determine their gene structures, conserved motifs and promoter. The non-synonymous/synonymous substitution ratios of paralogous and orthologous were less than 1, indicating that the GRF family mainly experienced purifying selection during evolution. According to the analysis of tissue-specific expression patterns, the participation of moso bamboo GRFs might be required during the formation and development of these five tissues. Moreover, PeGRF proteins might be involved in the regulation of plant development in biological processes. The qRT-PCR analysis demonstrated that *PeGRF* genes played essential roles in combating hormonal stresses and they might be involved in hormone regulation. PeGRF11, a nuclear localized protein as assessed by a subcellular localization assay, could interact with PeGIF3 in yeast and in planta according to yeast two-hybridization and bimolecular fluorescence complementation assays (BiFC) assays. But PeGRF11, as a TF, had no transcriptional activity in yeast. These results provide useful information for future functional research on the *GRF* genes in moso bamboo.

## Introduction

Bamboo is an important non-timber forest product worldwide, that financially supports nearly 250 million people (Peng et al., 2013). Moso bamboo, a widely cultivated bamboo species in China, possesses the greatest ecological, economic and cultural values (Liu et al., 2017; Peng et al., 2013). In China, moso bamboo is widely used for timber, paper, art wares and food (Wu et al., 2016), and its annual forest production has been estimated at approximately five billion US dollars (Wu et al., 2015).

Transcription factors (TFs) play essential roles in regulating plants growth and development, including metabolism, differentiation and reproduction. As DNA-binding proteins, TFs regulate gene expression by activating or inhibiting the mRNA transcription of multiple target genes (Filiz, Koç & Tombuloğlu, 2014). To date, many TFs have been identified in moso bamboo and other plants, including the TCP (Chai et al., 2017; Liu et al., 2018), PHD (Gao et al., 2017; Wang et al., 2015), MYB (Yang, Dai & Zhang, 2012; Cao et al., 2016b; Yang et al., 2019), NAC (Peng et al., 2015; Wang et al., 2016), WRKY (Cai et al., 2017; Li et al., 2017), HD-ZIP (Chen et al., 2017; Zhao et al., 2014) families, in which their members respond to hormones and participate in plant growth and development. Growth-regulating factor (GRF), a small plant-specific TF family, has been identified to contain two typical domains in the N-terminal region, QLQ (Gln, Leu, Gln; IPR014978) and WRC (Trp, Arg, Cys; IPR014977) (Cao et al., 2016a; Choi, Kim & Kende, 2004; Omidbakhshfard et al., 2015). The QLQ conserved region is also found in the yeast SWI2/SNF2 protein, which interacts with SNF11 to form a complex that participates in chromatin remodeling (Ma et al., 2017; Zhang et al., 2018). This protein–protein interaction domain, combines with the SNH domain of the GRF–interacting factor (GIF) family, which leads to the formation of the GRF–GIF complex involved in transcriptional activation (Kim, Choi & Kende, 2003). The WRC domain, a plant-specific domain, contains a putative zinc finger structural motif, which is thought to be involved in nuclear localization and DNA binding (Baloglu, 2014; Zhang et al., 2008). Furthermore, less-conserved motifs including FFD (Phe, Phe, Asp) and TQL (Thr, Gln, Leu) are remained in the C-termini of several GRF proteins (Kim, Choi & Kende, 2003; Ma et al., 2017).

In plants, functional analyses of GRFs have been reported, including in root, stem, and leaf development, flower and seed formation and environmental stress tolerance (Omidbakhshfard et al., 2015). *OsGRF1*, the first reported GRF member, may regulate gibberellic acid (GA)-induced stem elongation and transcriptional activity (Van der Knaap, Kim & Kende, 2000). The GRF proteins also participate in plant morphogenetic periods. In *Arabidopsis*, *AtGRF1* and *AtGRF2* overexpression lines show larger leaves and cotyledons, whereas the triple *AtGRF1*/*2*/*3* mutants produce smaller leaves and cotyledons based on the regulation of cell proliferation and cell expansion (Kim, Choi & Kende, 2003; Kim & Lee, 2006). *AtGRF4* has an important effect on the embryonic development of cotyledons and shoot apical meristem, and it is also involved in leaf development, owing to changes in cell numbers (Kim & Lee, 2006). Furthermore, *OsGRF4* may regulate panicle traits through its influence on the expression of the *CKX5* and *CKX1* genes, which are both cytokinin dehydrogenase precursor genes (Sun et al., 2016). The overexpression of *Brassica rapa* L. *GRF8* in *Arabidopsis* causes enlarged leaf organs owing to increased cell numbers (Wang et al., 2014). GIFs form functional complexes with GRFs by interacting with QLQ domains, which contribute to the regulation of plant growth and development (He et al., 2017; Kim & Tsukaya, 2015). For example, AtGRF5 cooperate with AtGIF1 to positively regulate the development of leaf primordia (Horiguchi, Kim & Tsukaya, 2005). Additionally OsmiR396d–OsGRF–OsGIF1 plays an important role in the development of inflorescences, which is beneficial to increase the rice yield (Liu et al., 2014). Similarly, Li et al. (2016) found that the miR396c–OsGRF4–OsGIF1 module effects grain size determination, which may increase the rice yield. Interestingly, ZmGRF10 plays a negative regulatory role in leaf size and plant height, but it may act as a fine-tuner in maintaining the regulatory functions of GRF–GIF complexes during cell proliferation (Wu et al., 2014). Moreover, *GRF* genes can also respond to abiotic stresses in plants. *AtGRF7* represses the expression of osmotic stress-responsive genes (including DREB2A) under normal conditions. However, the *atgrf7* mutant line enhances tolerances to salt and drought stresses compared with the wild-type and *AtGRF7*-overexpressing lines (Kim et al., 2012).

Here, we identified 18 moso bamboo *GRF* genes and investigated their phylogenetic relationships, motif organizations, gene structures, evolutionary divergence, promoter elements and expression profiles. Furthermore, we identified a possible functional gene, *PeGRF11*, which was performed subcellular localization and transcriptional activation assessments. It was then characterized using yeast two-hybridization and bimolecular fluorescence complementation assays (BiFC) assays.

## Materials and Methods

### Identification of the *GRF* gene family in moso bamboo

The GRF protein sequences of rice were collected from the Rice Genome Annotation Project (http://rice.plantbiology.msu.edu/) (Choi, Kim & Kende, 2004), and the genomic data of both maize and *Brachypodium distachyon* were downloaded from the Phytozome database (http://www.phytozome.net/) (Filiz, Koç & Tombuloğlu, 2014; Zhang et al., 2008). The moso bamboo genomic data were obtained through the National Center For Gene Research (NCGR) (http://server.ncgr.ac.cn/bamboo/index.php) to identify *PeGRF* genes. Based on Hidden Markov Model profiles, the QLQ (PF08880) and WRC (PF08879) domains from the Pfam database (http://pfam.janelia.org/) were employed to search against the moso bamboo genome database (Choi, Kim & Kende, 2004; Wheeler & Eddy, 2013). The SMART software was used to screen for candidate protein sequences containing the known conserved QLQ and WRC domains (Finn et al., 2016). Subsequently, the physicochemical parameters of the PeGRF proteins, such as the predicted number of amino acids, molecular weight and isoelectric point, were calculated using ExPASy (http://www.expasy.ch/tools/pi_tool.html). Additionally, the sequences of the PeGRF proteins were calculated using the DNAMAN (http://dnaman.software.informer.com/) algorithm.

### Phylogenetic tree, motif distribution and gene structure

A multiple sequence alignment was conducted using ClustalX 2.11 software with the default settings (Tamura, Nei & Kumar, 2004) (File S1). Based on the alignment, a phylogenetic tree was constructed using MEGA 6.0 software with the Neighbor-joining method and a bootstrap analysis of 1,000 replicates (Tamura et al., 2013). At the same time, we used RAxML software to construct a phylogenetic tree, and used the maximum likelihood method (ML) to analyze the data (Stamatakis, 2006). The *PeGRF* gene structures were analyzed using the online gene structure display server (GSDS 2.0, http://gsds.cbi.pku.edu.cn/) by comparing the genomic DNA sequences and corresponding coding sequences (CDSs) of individual genes (Guo et al., 2007). In addition, using the multiple em for motif elicitation (MEME) program (http://meme-suite.org/tools/meme), with the default parameters of a maximum number of 20 motifs and an optimum width of 6–200 residues, the motifs of PeGRF proteins were predicted.

### *Cis*-acting element analysis and gene ontology annotation

The promoter sequences, 2,000 bp regions upstream of the *PeGRF* translational start sites were downloaded from the bamboo genome database. The *cis*-regulatory elements from promoter sequences were analyzed using PlantCARE software (http://bioinformatics.psb.ugent.be/webtools/plantcare/html/) (Higo et al., 1999).

The protein sequences of PeGRFs were annotated using the Blast2GO program (https://www.blast2go.com/) (Conesa & Götz, 2008), and gene ontology (GO) terms were divided into three categories: molecular function, cellular component and biological process.

### Non-synonymous (Ka)/synonymous (Ks) substitution ratio analysis of homologous pairs

A BLASTN algorithm-based method was used in all-against-all nucleotide sequence similarity searches of three species (Altschul et al., 1997). To identify putative orthologs between two different species (A and B), each sequence from species A was searched against all sequences from species B, and then each sequence from species B was searched against all sequences from species A using BLASTN. If each of two sequences was the best hit to the other and they aligned over more than 300 bp, then they were defined as orthologs (Guo et al., 2014). When two *PeGRF* sequences were aligned over 300 bp and shared at least a 40% identity, the sequences were defined as paralogs. The results were used to calculate the Ka and Ks substitution rates by DnaSP version 5.10.1 (Librado & Rozas, 2009; Rozas, 2009). In addition, a sliding window analysis was performed with the following parameters: window length 150 bp and step size nine bp. The divergence time of the gene pairs was calculated using the formula *T* = Ks/2λ, where λ = 6.5 × 10^−9^ (Peng et al., 2013; Wang et al., 2017).

### Plant materials, growth conditions and hormone treatments

A total of 8-week-old moso bamboo seedlings, which grown in the greenhouse under long-day conditions of 16 h light and a continuous temperature of 25 ± 2 °C, were used for RNA isolation. The seedlings leaves were sprayed independently with 100 μM gibberellic acid (GA3), 100 μM methyl jasmonate (MeJA), and 100 μM abscisic acid (ABA) solutions and were collected at five time points (1, 3, 6, 12 and 24 h) after the hormone treatment (Khatun et al., 2017; Wu et al., 2016; Wang et al., 2017). The control groups (0 h) were unprocessed leaves before hormone treatments. The treatments were repeated three times and all samples were immediately frozen in liquid nitrogen and stored at −80 °C for further use.

### Expression profiling of moso bamboo *GRF* genes

The expression profile data were collected from the NCBI Short Read Archive database (https://trace.ncbi.nlm.nih.gov/Traces/sra/?study=ERP001341). The unprocessed BioProject ERP001341 RNA sequencing reads were trimmed to remove low quality base-calls (*Q* < 20) and clean adaptor sequences with the pipeline Fastq (Zhang et al., 2015). The paired clean reads were mapped to the *Phyllostachys edulis* reference genome using the pipeline tophat2, and cufflink, with defaults parameters, was used to detect differentially expressed genes (Trapnell et al., 2012). A heatmap of moso bamboo *GRF* genes was constructed using the Heatmapper Plus tool (http://bar.utoronto.ca/ntools/cgi-bin/ntools_heatmapper_plus.cgi) (Toufighi et al., 2005), which included five different tissues or developmental stages.

### RNA extraction and qRT-PCR analysis

Total RNA was extracted using an RNAprep Pure Plant Kit (Tiangen, Beijing, China), and the quality was checked by 1.5% agarose gel electrophoresis. Then, the extracted RNAs were reverse transcribed to cDNAs using a Prime-ScriptRT Reagent Kit (TaKaRa, Kusatsu, Japan) in accordance with the manufacturer’s instructions. Gene-specific primers were designed using Primer Premier 5.0, and used for qRT-PCR, which was performed as follows: 95 °C for 30 s; followed by 40 cycles of 95 °C for 10 s, 55 °C for 15 s and 72 °C for 10 s. The tonoplast intrinsic protein 41 gene acted as an internal control (Fan et al., 2013).

### Subcellular localization and transcriptional activation

The full-length CDS of *PeGRF11* was cloned using the designed primers (Table S1) and was identified by sequencing. Subsequently, the amplified PCR products were digested with *Xba* I and *Bam*H I restriction enzymes and independently inserted into the pCAMBIA1305-GFP vector containing CaMV35S promoter. These constructs were transformed into *Agrobacterium tumefaciens* EHA105. Then, *Agrobacterium* independently harboring the 35S::PeGRF11::GFP and 35S::GFP control vectors were infiltrated into *Nicotiana benthamiana* leaves using the injection method. The transient expression was examined using a fluorescence microscope (CarlZeiss LSM710; Jena, Germany) after transfected plants had been maintained in the dark for 36–48h.

The cDNA sequences of *PeGRF11* were cloned into the pGBKT7 vector using *Eco*R I and *Bam*H I restriction sites. The co-transform vector (pGBKT7-53 and pGADT7-T) and the empty vector (pGBKT7) were used as positive and negative controls, respectively. The plasmids pGBKT7::PeGRF11, pGBKT7-53+pGADT7-T and the empty vector (pGBKT7) carrying the GAL4 DNA-binding domain and TRP1 nutritional marker were independently transformed into yeast strain Y2HGold. The specific primers were listed in Table S1.

### Yeast two-hybridization and BiFC assays

PeGRF11 was cloned into the pGBKT7 bait vector, and the CDS of PeGIF3 was cloned into the pGADT7 vector. Primer sequences were listed in Table S1. Recombinant plasmids were cotransformed into yeast strain AH109 and then plated on selective media (SD/−Leu/−Trp and SD/−Leu/−Trp/−His/−Ade/X-α-GAL) to screen for positive clones (Zeng et al., 2018).

To obtained expression vectors (pFGC-N-YFP and pFGC-C-YFP), eYFP fragments coding for the N-terminal 173 aa and C-terminal 155 aa were cloned into pFGC5941 (Hu et al., 2013). The CDSs of PeGRF11 and PeGIF3 were introduced into pFGC-N-YFP and pFGC-C-YFP, respectively, resulting in N-terminal in-frame fusions with N-YFP and C-terminal in-frame fusions with C-YFP, respectively (Table S1). The vector constructs were transformed into *Agrobacterium tumefaciens* and injected into the leaves of tobacco (*N. benthamiana*) (Waadt & Kudla, 2008). Images of fluorescence and 4,6-diamidino-2-phenylindole staining of transfected plants were taken using a confocal laser scanning microscope after a 36–48 h dark treatment.

### Statistical analysis

Statistical Product and Service Solutions (SPSS) software was used for analyzing the statistically significant differences. All values were presented as means ± standard deviations of three replicates, and significance levels were determined as **P < 0.01 and *P < 0.05.

## Results

### Identification and sequence analyses of moso bamboo *GRF* genes

A BLASTP analysis-based search was performed against the moso bamboo genome database using the consensus protein sequences from the Hidden Markov Model profile as a query. Gene identifier of the GRFs in the NCGR database was provided (Table 1). For easily research, we named the genes PeGRF1–18 in accordance with their scaffold positions (Table 1). The CDS lengths of the *PeGRF*s varied slightly: the longest was *PeGRF12* (1,656 bp) and the shortest was PeGRF11 (513 bp) (Table 1; Files S2 and S3). The encoded PeGRF proteins ranged from 170 (PeGRF11) to 551 (PeGRF12) amino acids in length, with an average molecular weight of 39,250.5 Da, and the isoelectric points of which varied from 4.92 to 9.57 (Table 1). The details of the *GRF* genes in rice, maize and *Brachypodium distachyon* were listed in Table S2.

**Table 1:** Detailed information of *GRF* genes in moso bamboo.

Name	Gene identifier*	Scaffold	Location coordinates (5′-3′)	ORF length (bp)	Protein			
					Length (aa)	PI	Mol.Wt. (Da)	Exons
PeGRF1	PH01000005G1660	PH01000005	1047802–1052107 (−stand)	1,170	389	9.14	41,978.03	4
PeGRF2	PH01000045G1330	PH01000045	869928–873464 (+stand)	1,008	335	9.04	37,271.77	3
PeGRF3	PH01000083G1570	PH01000083	981150–986331 (−stand)	1,038	345	9.16	37,728.96	3
PeGRF4	PH01000152G0440	PH01000152	299152–308034 (−stand)	1,407	468	6.25	51,266.89	6
PeGRF5	PH01000197G1460	PH01000197	930248–936852 (+stand)	1,179	392	7.71	42,011.71	4
PeGRF6	PH01000387G0730	PH01000387	652447–654934 (+stand)	1,362	453	6.73	49,141.86	3
PeGRF7	PH01000513G0530	PH01000513	390562–394641 (+stand)	1,206	401	8.34	43,195.84	7
PeGRF8	PH01000842G0540	PH01000842	394216–395977 (+stand)	639	212	9.3	22,503.55	3
PeGRF9	PH01001096G0260	PH01001096	234426–237322 (+stand)	1,113	370	6.21	40,368.06	3
PeGRF10	PH01001291G0040	PH01001291	25862–27620 (−stand)	939	312	8.47	34,018.72	2
PeGRF11	PH01001304G0330	PH01001304	285822–288315 (−stand)	513	170	9.32	18,478.01	2
PeGRF12	PH01001605G0350	PH01001605	262844–268296 (+stand)	1,656	551	8.63	58,800.40	8
PeGRF13	PH01002169G0420	PH01002169	248859–249908 (+stand)	651	216	9.57	22,808.98	3
PeGRF14	PH01002618G0290	PH01002618	154780–159948 (+stand)	1,335	444	6.72	48,549.03	5
PeGRF15	PH01002701G0020	PH01002701	4592–8886 (−stand)	783	260	4.92	27,787.69	3
PeGRF16	PH01003592G0180	PH01003592	111903–116228 (+stand)	1,239	412	9.29	46,690.54	4
PeGRF17	PH01005386G0020	PH01005386	5903–9192 (+stand)	1,236	411	9.3	46,270.16	4
PeGRF18	PH01087379G0010	PH01087379	32–1145 (−stand)	996	331	8.24	36,416.32	2

**Note:**

* Gene identifier represents the gene name in the NCGR.

**Figure 1:**
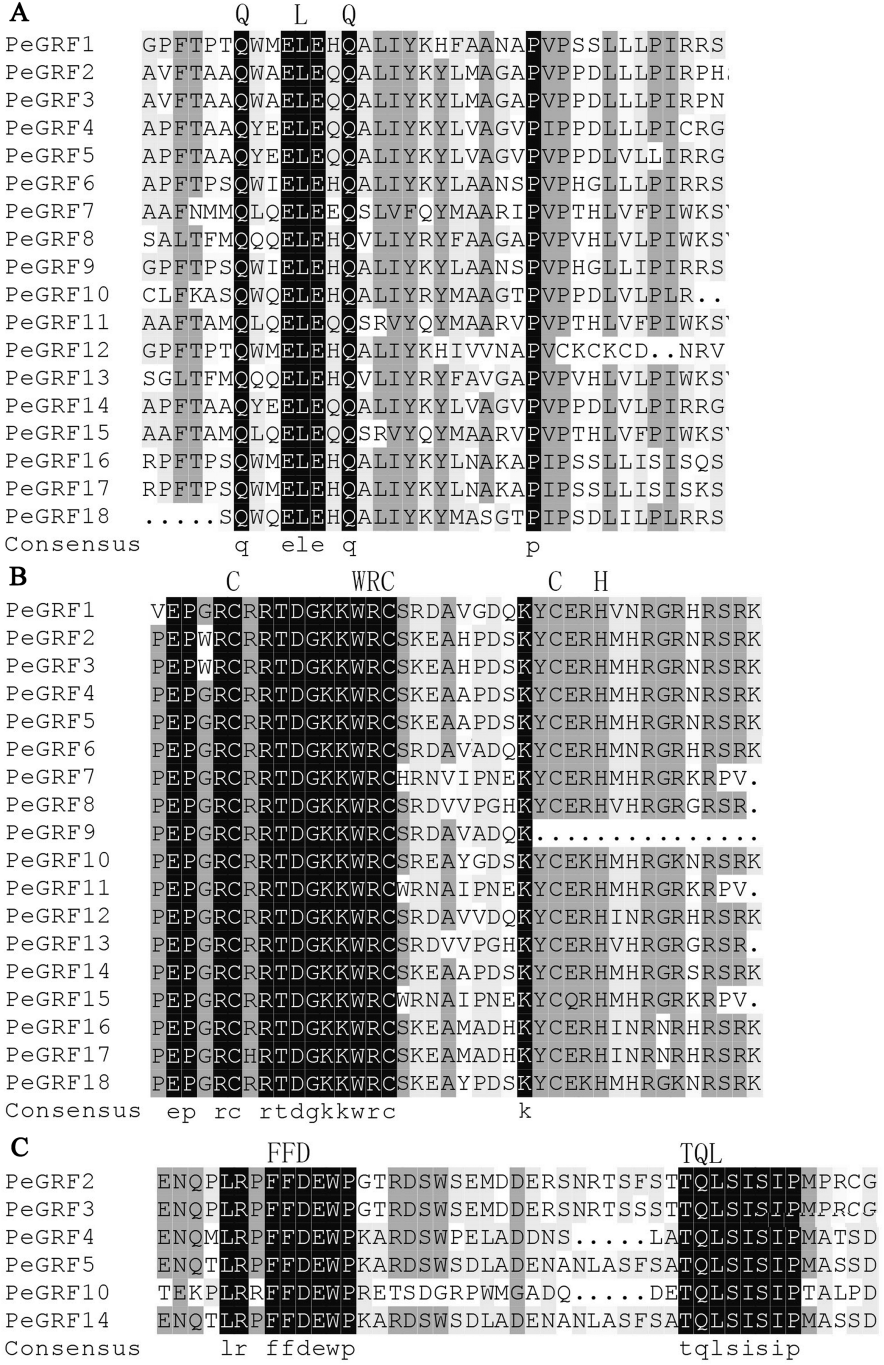
Sequence alignment of the conserved domains in PeGRF proteins. (A) The QLQ domains of PeGRF proteins. (B) The WRC domains of PeGRF proteins containing the zinc finger Cys3His motif. (C) The FFD and TQL motifs in the C-termini of PeGRF proteins.

To further investigate the characteristics of *PeGRF*s, 18 putative proteins were aligned using DNAMAN software. All PeGRF proteins contained the highly conserved QLQ and WRC domains in their N-terminal regions (Figs. 1A and 1B). Moreover, zinc finger motifs were found within the WRC amino acid stretch (Fig. 1A). Additionally, the FFD and TQL motifs, two smaller stretches of amino acid residues, were found in the C-terminal regions of PeGRF2, -3, -4, -5, -10 and -14 (Fig. 1C).

### Phylogenetic analysis of GRF proteins

**Figure 2:**
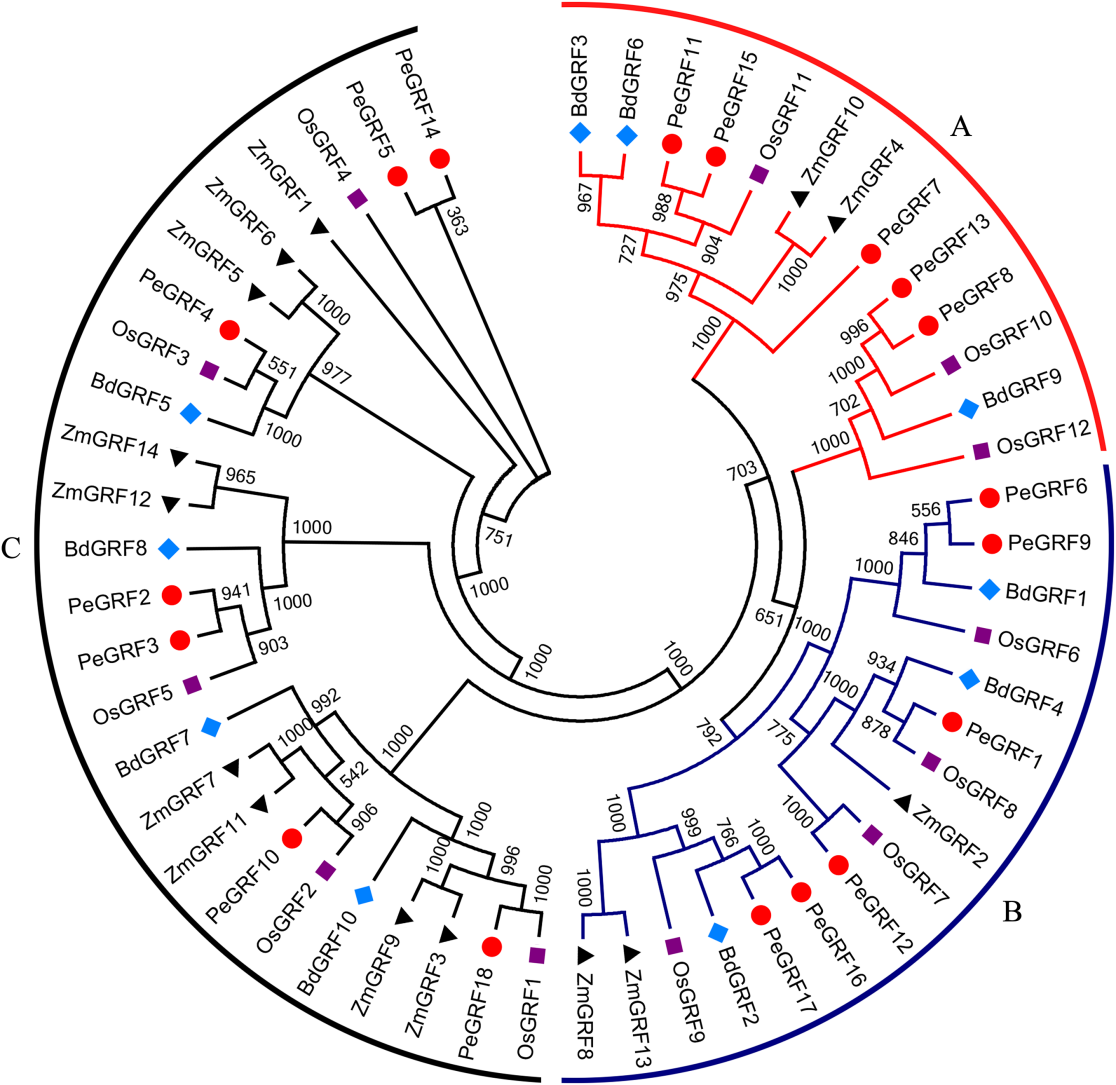
Phylogenetic analysis of GRF from moso bamboo, rice, maize and *Brachypodium distachyon*. The unrooted Neighbor-joining tree was constructed using MEGA 6, with 1,000 replications of boot strap values. Different shapes and colors represented four species.

To evaluate the evolutionary relationships of GRFs between moso bamboo and other species including rice, maize and *Brachypodium distachyon*, the phylogenetic tree of 54 GRF motif-containing proteins was constructed based on the multiple sequence alignment. As shown in Fig. 2, GRF proteins were divided into three subfamilies on the basis of previous studies in rice and maize (Zhang et al., 2008). Subsequently, we performed a ML phylogenetic tree and found that the *GRF* genes were divided into the same three subfamilies consistent with the neighbor-joining tree (Fig. S1). Moso bamboo GRFs were relatively evenly distributed among the three subfamilies (A, B and C) (Fig. S2).

### Conserved motifs and gene structure analyses of moso bamboo *GRF* genes

**Figure 3:**
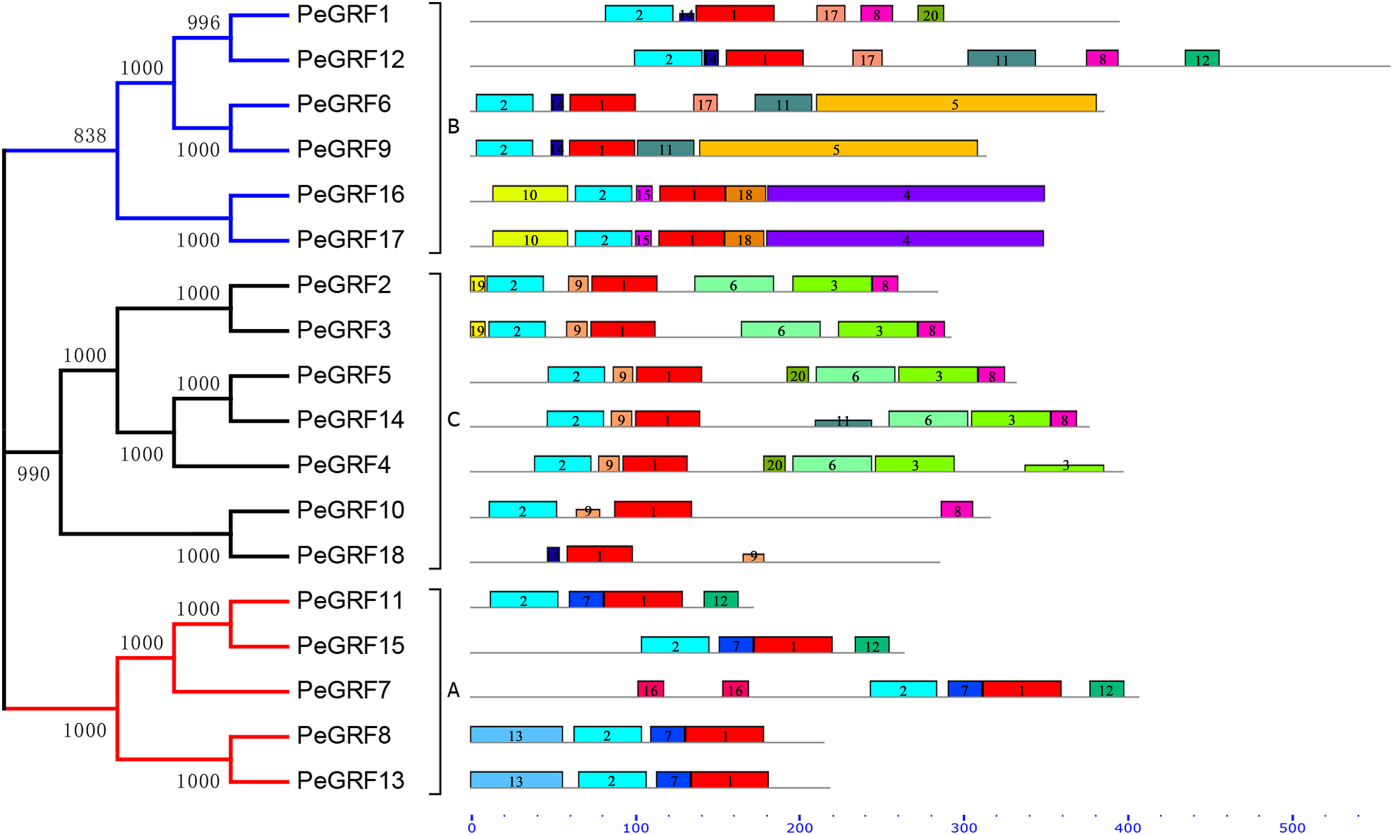
Conserved motifs of PeGRF proteins. The conserved motifs of 18 PeGRFs were detected using MEME. The lengths of 20 different motifs were shown in proportion and accompanied by their phylogenetic relationships. These putative motifs were represented by boxes with different colors and numbers and were listed in Table S3.

Conserved motifs and intron/exon arrangements were analyzed to further explore the structural diversity of moso bamboo GRFs. In total, 20 distinct motifs in PeGRFs were predicted by the MEME program and were annotated using Pfam online tools. The details of 20 conserved domains, best possible matches and motif lengths were listed in Table S3. Motif 1 and 2 were respectively encoded in WRC and QLQ domains, which were two specific conserved regions of GRF proteins, but the remaining 18 motifs were not. Motif 2 was present in all PeGRFs, except PeGRF18, which was similar to BrGRF (Wang et al., 2014). Interestingly, *PeGRF* genes of the same subgroup possessed similar motifs. For example, most members from subfamily C contained motifs 1, 2, 3, 6, 8 and 9, and they maintained relatively stable structure. The subfamily A also contained similar motif types (Fig. 3).

**Figure 4:**
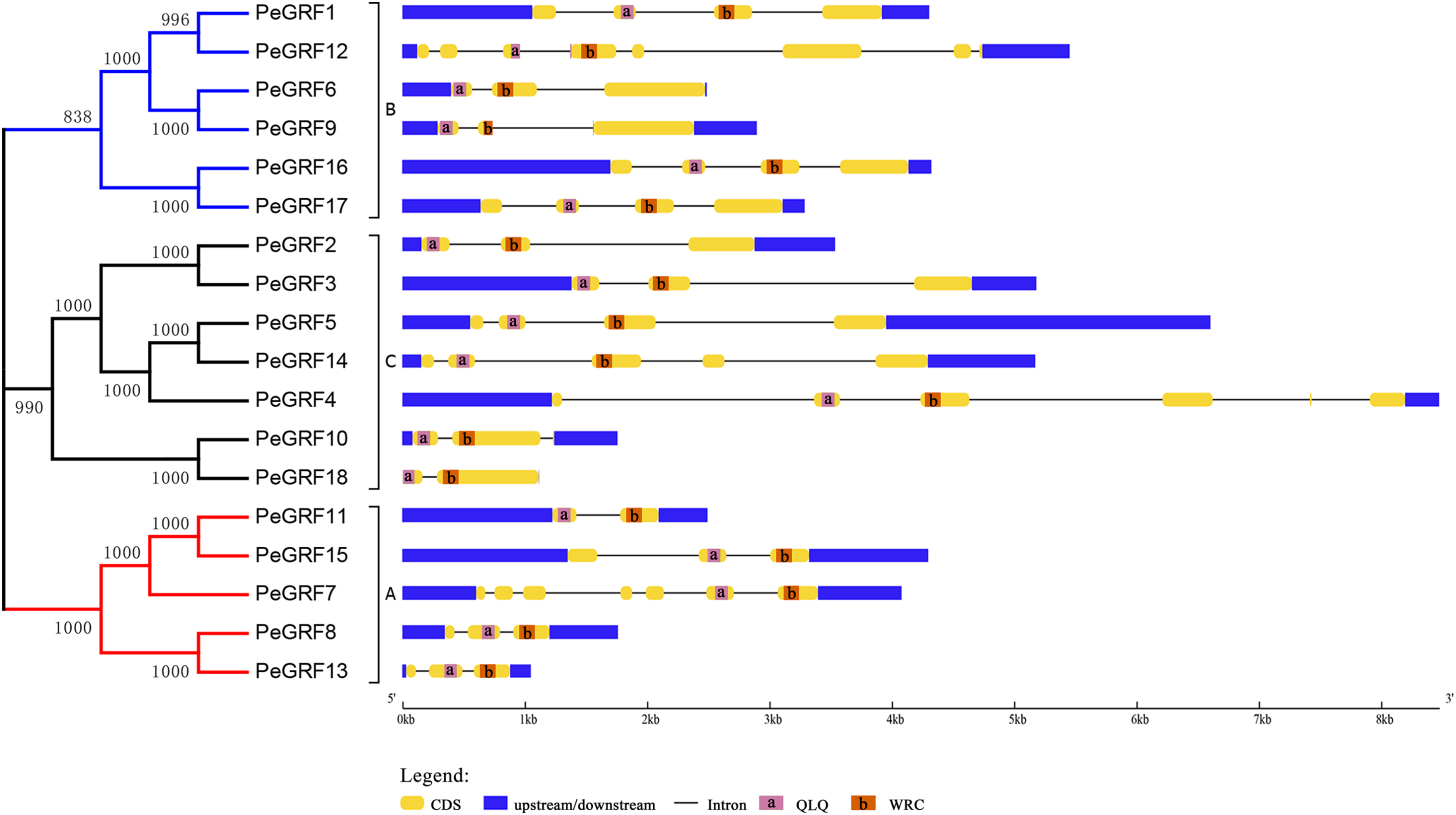
Gene structure analysis of *GRF* genes in moso bamboo. The gene structure was shown three parts, upstream/downstream, exons and introns, which were represented by blue boxes, yellow boxes and black lines, respectively. In the exon structure, QLQ and WRC domains were denoted by purple and orange boxes with letters, respectively.

Subsequently, the intron/exon organizations of *PeGRF*s were analyzed using the GSDS website to deduce the possible structural evolution. The 18 *PeGRF*s had different numbers of exons, ranging from two to eight (Table 1). Most of *PeGRF* (*PeGRF2*, *-3*, *-6*, *-8*, *-9*, *-13* and *-15*) genes had three exons, followed by four (four out of 18) and three (three out of 18) exons (Fig. 4). The intron/exon structures of the 10 paralogous *GRF* gene pairs were further investigated, and six pairs had been altered. Among them, *PeGRF5*, *-11* and *-14* had one less intron compared with *PeGRF14*, *-15* and *-4*, respectively. However, other homologous pairs had greater differences in the numbers of introns. Thus, the gain and loss of introns during the evolutionary period might result in the structural diversity found among *PeGRF* genes (Cao et al., 2016a).

### Evolutionary patterns of *GRF* genes

**Figure 5:**
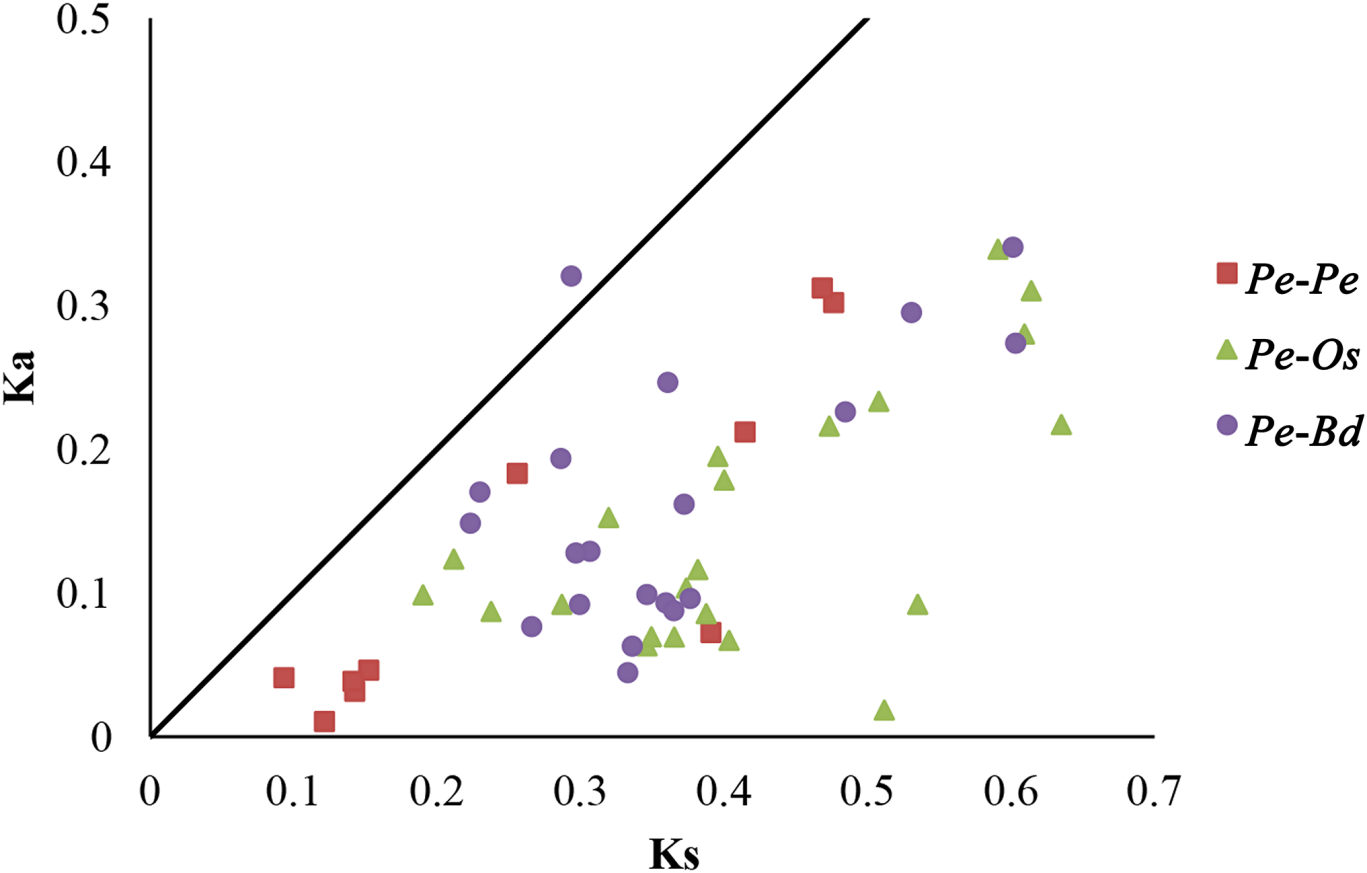
Distribution of Ka and Ks from paralogous (*Pe*-*Pe*) and orthologous (*Pe*-*Os* and *Pe*-*Bd*) gene pairs. Different shapes and colors represented homologous gene pairs of *Pe*-*Pe, Pe*-*Os* and *Pe-Bd*, respectively, and the black line indicated the slope of Ka/Ks = 1.

Selective pressure on CDSs were assessed using Ka/Ks ratios, in which Ka/Ks ratios greater than 1, equal to 1 and less than 1 indicated positive, neutral and negative or purifying selection, respectively (Juretic et al., 2005). To further understand the evolutionary selection pressure on the *GRF* genes in three plant species, 10 pairs of paralogs in moso bamboo (*Pe*-*Pe*), 22 orthologous pairs between moso bamboo and rice (*Pe*-*Os*), and 20 orthologous pairs between moso bamboo and *Brachypodium distachyon* (*Pe*-*Bd*) were screened by bidirectional best-hit analyses (Table 2; Table S4). The Ka/Ks ratios of all homologous gene pairs were less than 1, except *PeGRF10/BdGRF7* (Fig. 5), indicating that the *GRF genes* of these species had mainly undergone strong purifying selection. Remarkably, six paralogous gene pairs of moso bamboo had Ka/Ks ratios of less than 0.5, and the maximum ratio was only 0.716. The Ka/Ks ratios of 31 orthologous pairs (*Pe*-*Os* and *Pe*-*Bd*) were less than 0.5. In addition, the relative Ks values varied between 0.086 and 0.716 for paralogous pairs, deducing that the divergence times of moso bamboo *GRF* genes were concentrated between 7.18 and 36.65 million years ago. For orthologous genes, *Pe*-*Os* and *Pe*-*Bd*, the Ks values were centrally distributed between 0.2–0.45 and 0.2–0.4, respectively, which indicated that moso bamboo diverged from rice and *Brachypodium distachyon* 15.38–34.62 and 15.38–30.77 million years ago, respectively. Consequently, we hypothesized that the moso bamboo GRF family underwent a duplication event after its divergence from rice and *Brachypodium distachyon* (Peng et al., 2013). Sliding-window analyses were also performed to more clearly understand the Ka/Ks ratios of homologous pairs (Figs. S3, S4 and S5).

**Table 2:** Estimated divergence times for *GRF* gene pairs in moso bamboo.

*Pe-Pe*	Ka	Ks	Ka/Ks	Duplication date (million years)
*PeGRF9/PeGRF6*	0.04615	0.15242	0.303	11.72461538
*PeGRF8/PeGRF13*	0.03147	0.14266	0.221	10.97384615
*PeGRF7/PeGRF15*	0.3019	0.47649	0.634	36.65307692
*PeGRF7/PeGRF11*	0.07237	0.391	0.185	30.07692308
*PeGRF5/PeGRF14*	0.18311	0.25592	0.716	19.68615385
*PeGRF4/PeGRF5*	0.21185	0.4148	0.511	31.90769231
*PeGRF4/PeGRF14*	0.31207	0.4686	0.666	36.04615385
*PeGRF3/PeGRF2*	0.04093	0.09334	0.438	7.18
*PeGRF17/PeGRF16*	0.03841	0.14145	0.272	10.88076923
*PeGRF11/PeGRF15*	0.01046	0.12157	0.086	9.351538462

### Gene ontology annotation

**Figure 6:**
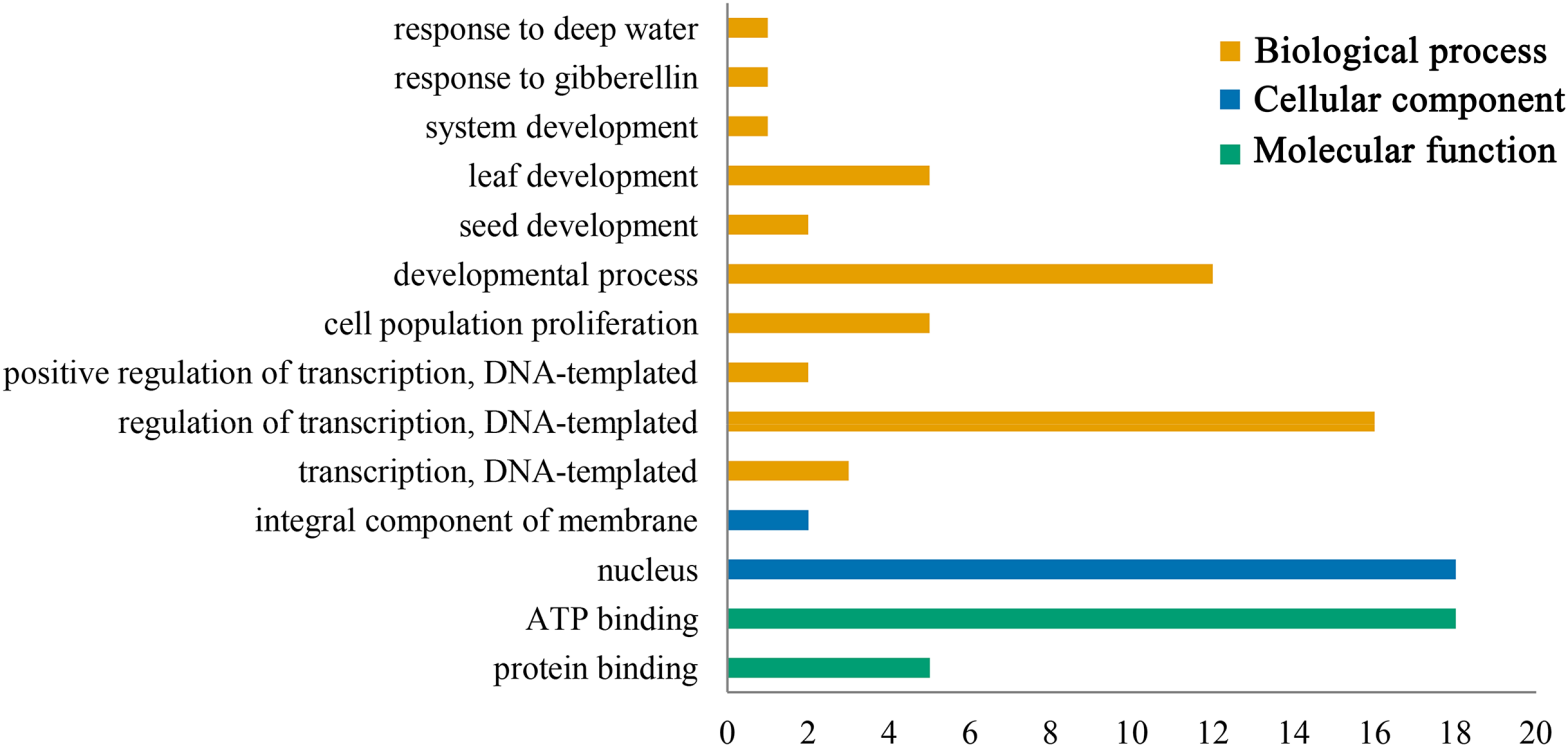
Gene ontology (GO) annotation of PeGRF protein. Bars indicated the number of genes with the same term.

Gene ontology functional annotation analysis indicated that ATP binding (GO:0005524) and protein binding (GO:0005515) were enriched in the molecular function category, and the PeGRFs showed protein binding components, which was related to the GRF family protein binding properties. The classification of cellular component showed that all PeGRF proteins were classified as nuclear-related components (GO:0005634), indicating that the moso bamboo GRFs could act as nuclear localization TFs. Additionally, the PeGRFs were involved in 10 different biological processes. In the biological process category, developmental process (GO:0032502) and regulation of transcription, DNA-templated (GO:0006355) were the most abundant terms, both greater than 60% (Fig. 6; Table S5).

### Promoter analysis

**Figure 7:**
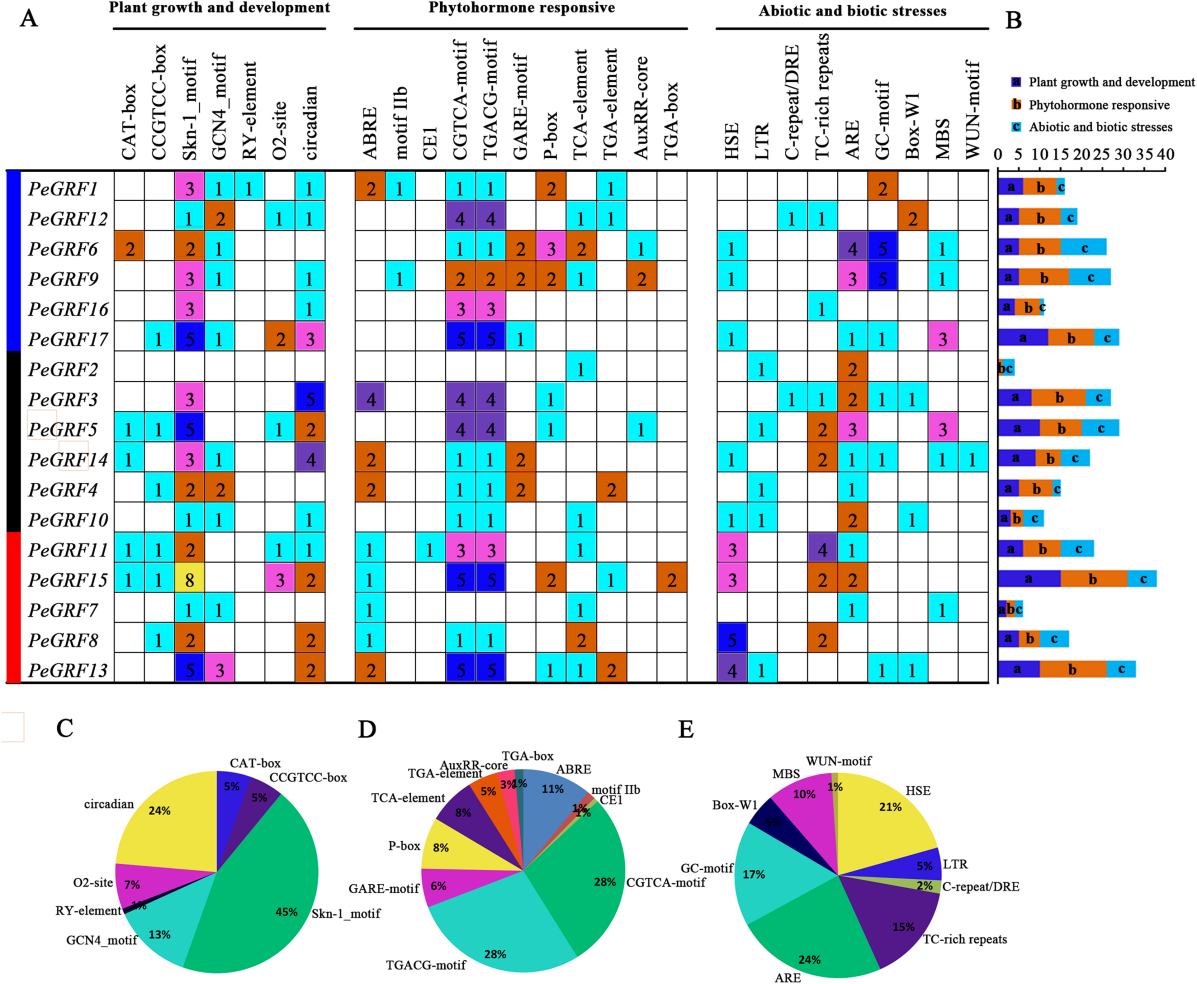
*Cis*-acting element analysis of moso bamboo *GRF* genes. (A) The investigation of the numbers of promoter elements in the *PeGRF* promoter regions. (B) Statistics for the number of the promoter elements in three major subfamilies, which were indicated by different colored boxes with letters. (C–E) The proportion of each promoter in the category.

The promoter regions 2,000 bp upstream of 17 *PeGRF* genes were surveyed and this helped us better understand the potential functions and regulatory mechanisms of *GRF* genes in moso bamboo (Table S6). *PeGRF18* was excluded because it did not contain a promoter sequence. The *cis*-elements of *PeGRF*s were divided into three major functional categories, plant growth and development, phytohormone responsive, and abiotic or biotic stress (Fig. 7). The Skn-1 and GCN4 motifs, involved in endosperm expression, were found 49 and 14 times, respectively, and accounted for 58% of the plant growth and development-related motifs. The CAT-box and CCGTCC-box *cis*-elements, which were involved in meristem expression, were detected in the promoters of the *PeGRF*s, and circadian control elements were also found. Most of *PeGRF* genes contained MeJA-responsiveness elements, CGTCA and TGACG motifs, which appeared 82 times, representing 56% of the hormone-related *cis*-acting elements. The GARE and P-box motifs, GA-responsive elements, were observed in five and seven *PeGRF* genes, respectively, which represented 14% of the hormone-responsive elements of *PeGRF*s. Moreover, ABA-responsive element (ABRE, motif IIb and CE1), SA-responsive element (TCA-element), and uxin-responsive element (TGA-element, AuxRR-core, and TGA-box) were presented in 10, nine and eight *PeGRF* genes, respectively. In addition, some abiotic and biotic stress-related *cis*-acting elements were also identified in the putative promoter regions of the *PeGRF* genes. Heat-stress-responsiveness elements and low-temperature-responsiveness elements (ARE and GC motifs) appeared 20 and 39 times, accounting for 21% and 40% of the stress-related *cis*-acting elements, respectively. However, *cis*-acting elements related to fungal elicitor-responsive (Box-W1) and wound-responsive (WUN motif) elements were only identified in four and one genes, respectively.

### Expression profiles of *PeGRF* genes

**Figure 8:**
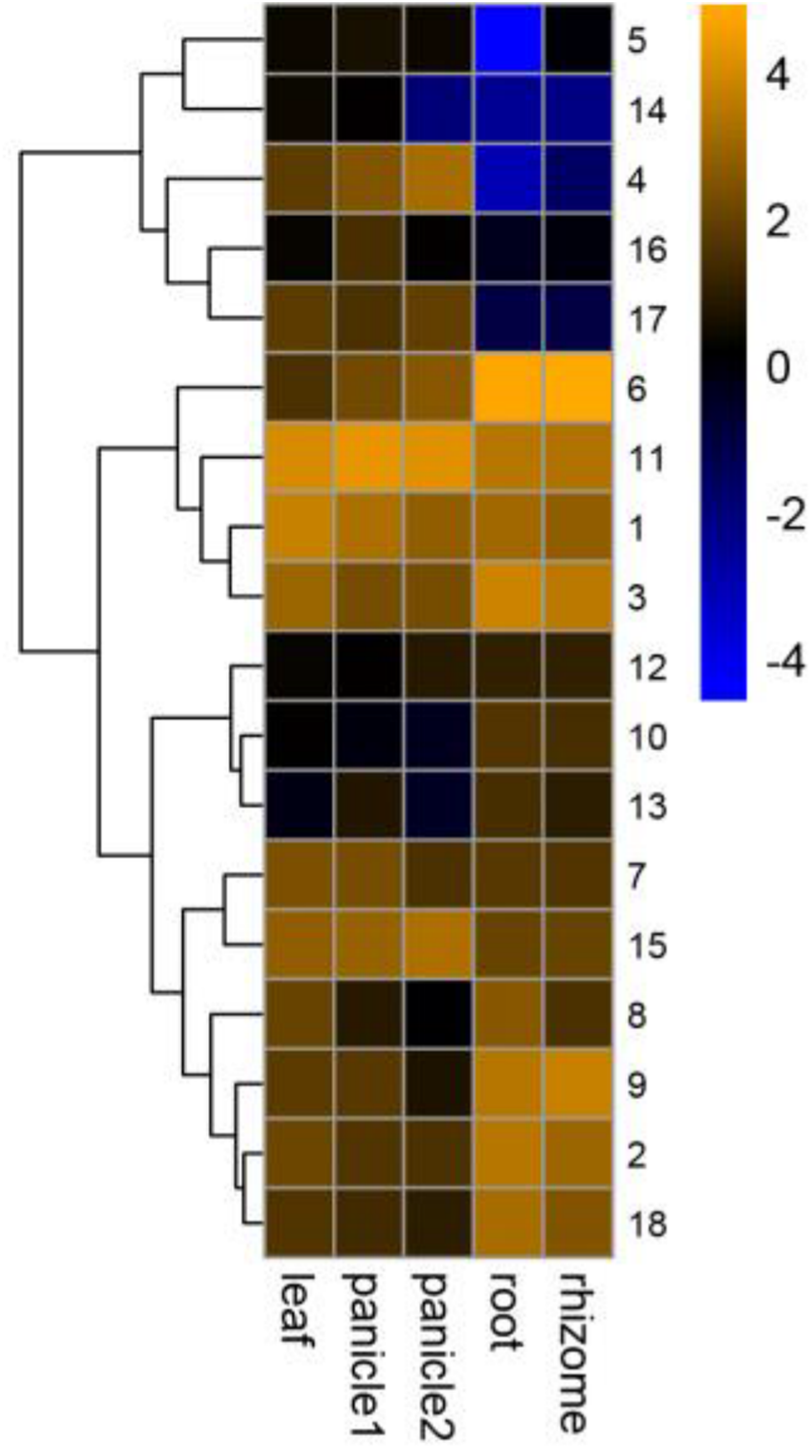
Expression profiles of *PeGRF* genes in different tissues and developmental stages: leaf, early panicle (panicle 1), advanced panicle (panicle 2), root and rhizome. The heatmap showed the hierarchical clustering of 18 *GRF* genes in different tissues. Blue and orange indicated low and high levels respectively, of transcript abundance.

The GRF family was involved in the development of plant organs (Khatun et al., 2017; Wang et al., 2014; Wu, Wang & Zhuang, 2017). Different *PeGRF* genes were detected in different tissues (Fig. 8; Table S7). Ten moso bamboo genes (*PeGRF1*, -*2*, -*3*, -*6*, -*7*, -*8*, -*9*, -*11*, -*15* and -*18*) exhibited high expression levels in five tissues or developmental stages, suggesting that these genes were most likely involved in the formation of these tissues. Subsequently, four paralogous pairs (*PeGRF4/PeGRF5*, *PeGRF4/PeGRF14*, *PeGRF8/PeGRF13* and *PeGRF5/PeGRF14*) showed distinct expression patterns, implying that duplicated genes’ functions might have differentiated. Notably, all moso bamboo *GRF* genes were highly expressed in leaf, with the exception of *PeGRF13*, potentially indicating that the *GRF* gene family played an important role in periods of leaf growth and development.

### Expression analysis of *PeGRF* genes exposed to hormone treatments

**Figure 9:**
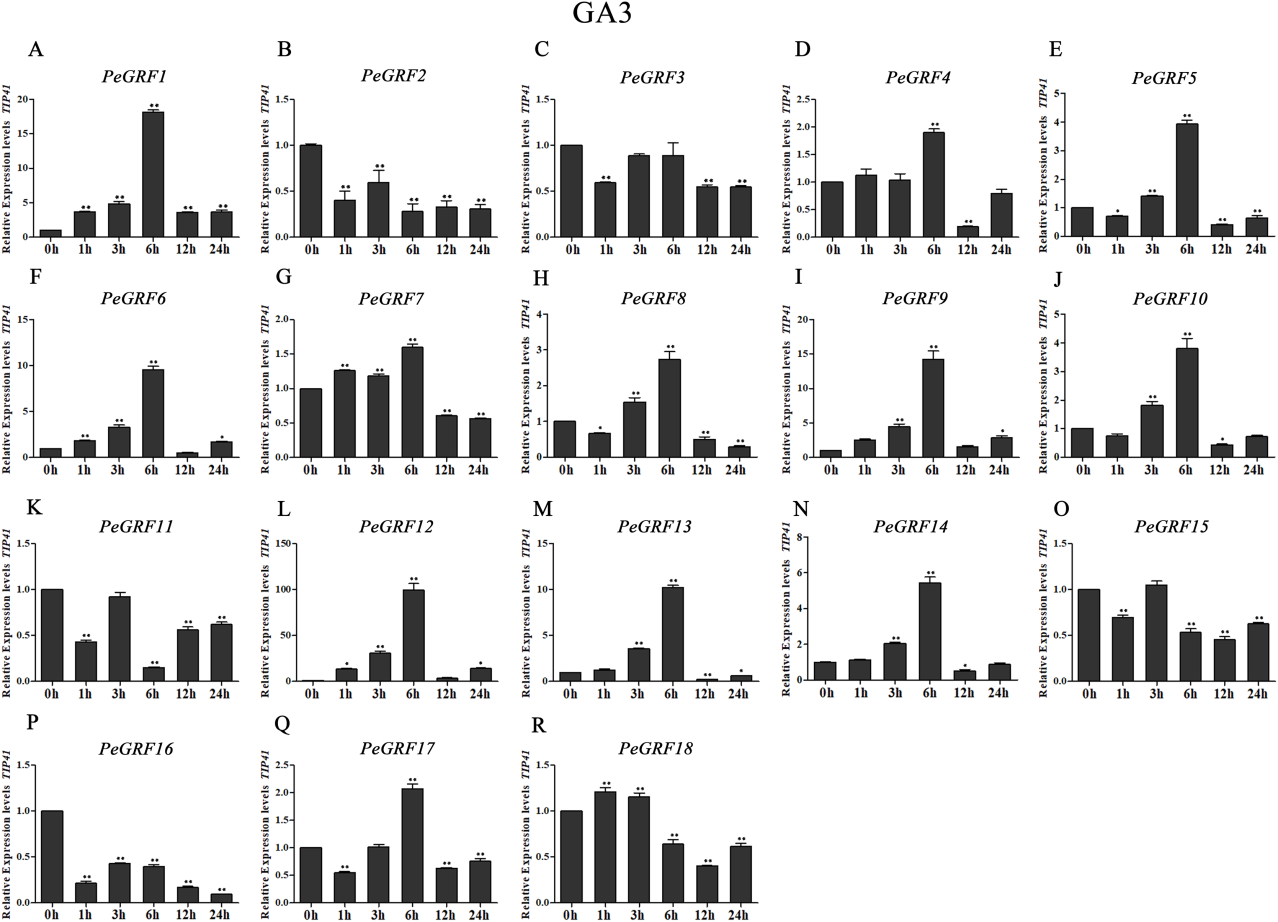
Quantitative RT-PCR analysis of the *PeGRF* genes in moso bamboo in response to GA3 stress. (A–R) *PeGRF1*–*PeGRF18*. The expression level of the 0-h treatment time point was defined as 1, and the levels at other time points were shown as relative fold changes compared with the control (0 h). The experiments were performed with three biological and three technical replicates, obtaining mean values and SDs. Significant differences in the expression level were indicated by asterisks (*P < 0.05, **P < 0.01).

**Figure 10:**
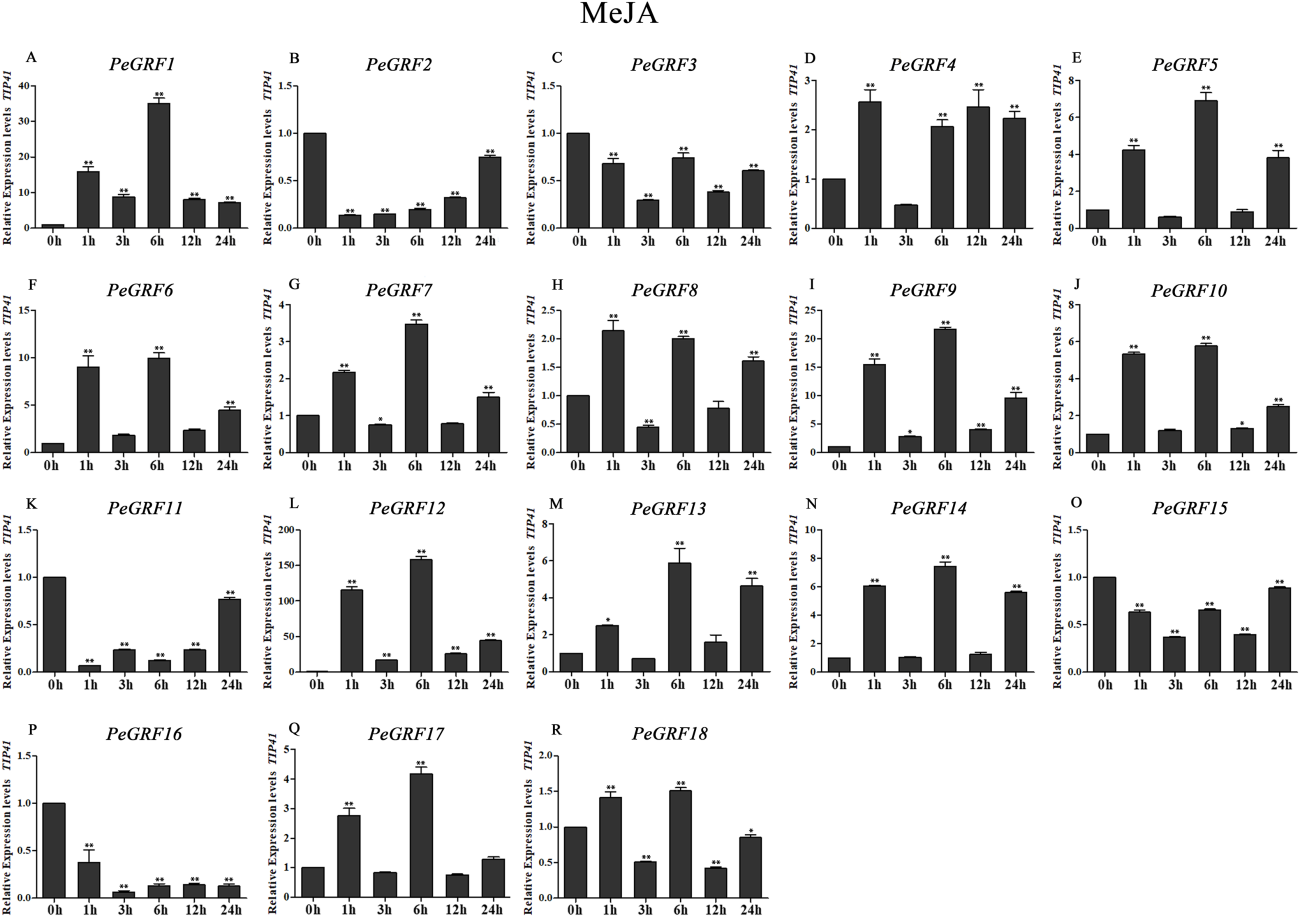
Quantitative RT-PCR analysis of the *PeGRF* genes in moso bamboo in response to MeJA stress. (A–R) *PeGRF1*–*PeGRF18*. The expression level of the 0-h treatment time point was defined as 1, and the levels at other time points were shown as relative fold changes compared with the control (0 h). The experiments were performed with three biological and three technical replicates, obtaining mean values and SDs. Significant differences in the expression level were indicated by asterisks (*P < 0.05, **P < 0.01).

**Figure 11:**
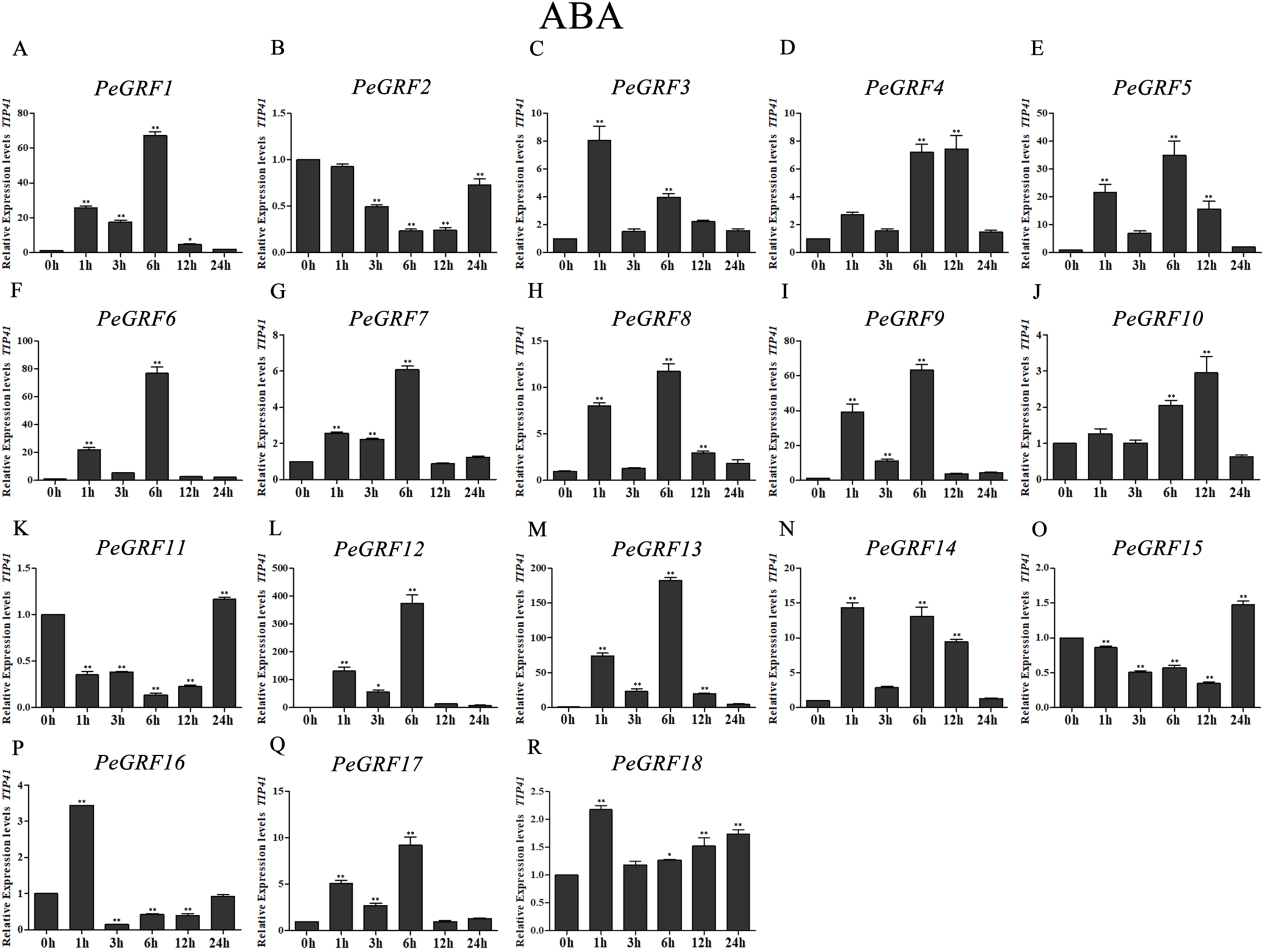
Quantitative RT-PCR analysis of the *PeGRF* genes in moso bamboo in response to ABA stress. (A–R) *PeGRF1*–*PeGRF18*. The expression level of the 0-h treatment time point was defined as 1, and the levels at other time points were shown as relative fold changes compared with the control (0 h). The experiments were performed with three biological and three technical replicates, obtaining mean values and SDs. Significant differences in the expression level were indicated by asterisks (*P < 0.05, **P < 0.01).

Plant hormones play vital roles in plant growth and development. Furthermore, *OsGRF1*, discovered 18 years ago, was predicted to be a Gibberellin 3 (GA3)-induced gene (Van der Knaap, Kim & Kende, 2000). To investigate whether moso bamboo *GRF* genes had roles in response to phytohormones, qRT-PCR was performed on the 18 *PeGRF* genes in moso bamboo leaves 0, 1, 3, 6, 12 and 24 h after hormone treatments. The specific primers for moso bamboo *GRF* genes were listed in Table S8. The transcriptional levels of the 18 *PeGRF* genes exhibited different degrees of change after hormone treatments (Figs. 9, 10 and 11).

A total of 13 *PeGRF* genes showed up-regulated expression levels in response to the GA3 treatment. In particular, the expression levels of 12 genes peaked at 6 h, with the exception of *PeGRF18* (Fig. 9). *PeGRF1*, *-9* and *-12* exhibited strongly up-regulated levels at five time points compared with at 0 h (Figs. 9A, 9I and 9L). Additionally, *PeGRF13* and *PeGRF14* were respectively up-regulated by 10-fold and fivefold at 6 h compared with at 0 h and were then down-regulated (Figs. 9M and 9N). *PeGRF6* was only down-regulated at 12 h by the GA3 treatment and was up-regulated by ninefold at 6 h (Fig. 9F). In contrast, the expression levels of three members (*PeGRF2*, *-11* and *-16*) were prominently down-regulated after the GA3 treatment (Figs. 9B, 9K and 9P).

In total, nine of 18 *PeGRF* genes (*PeGRF1*, *-5*, *-6*, *-9*, *-10*, *-12*, *-13*, *-14* and *-17*) were strongly up-regulated over fourfold by the MeJA treatment, while three genes (*PeGRF2*, *-11* and *-16*) were down-regulated to less than 0.5-fold compared with the control group (0 h) (Fig. 10).

Moso bamboo *GRF* genes showed different responses following the ABA treatment (Fig. 11), with 12 genes being significant up-regulated more than fourfold compared with the control (0 h). Notably, *PeGRF11* and *PeGRF15* had comparatively lower transcriptional levels after the ABA treatment.

### Subcellular localization of PeGRF11

**Figure 12:**
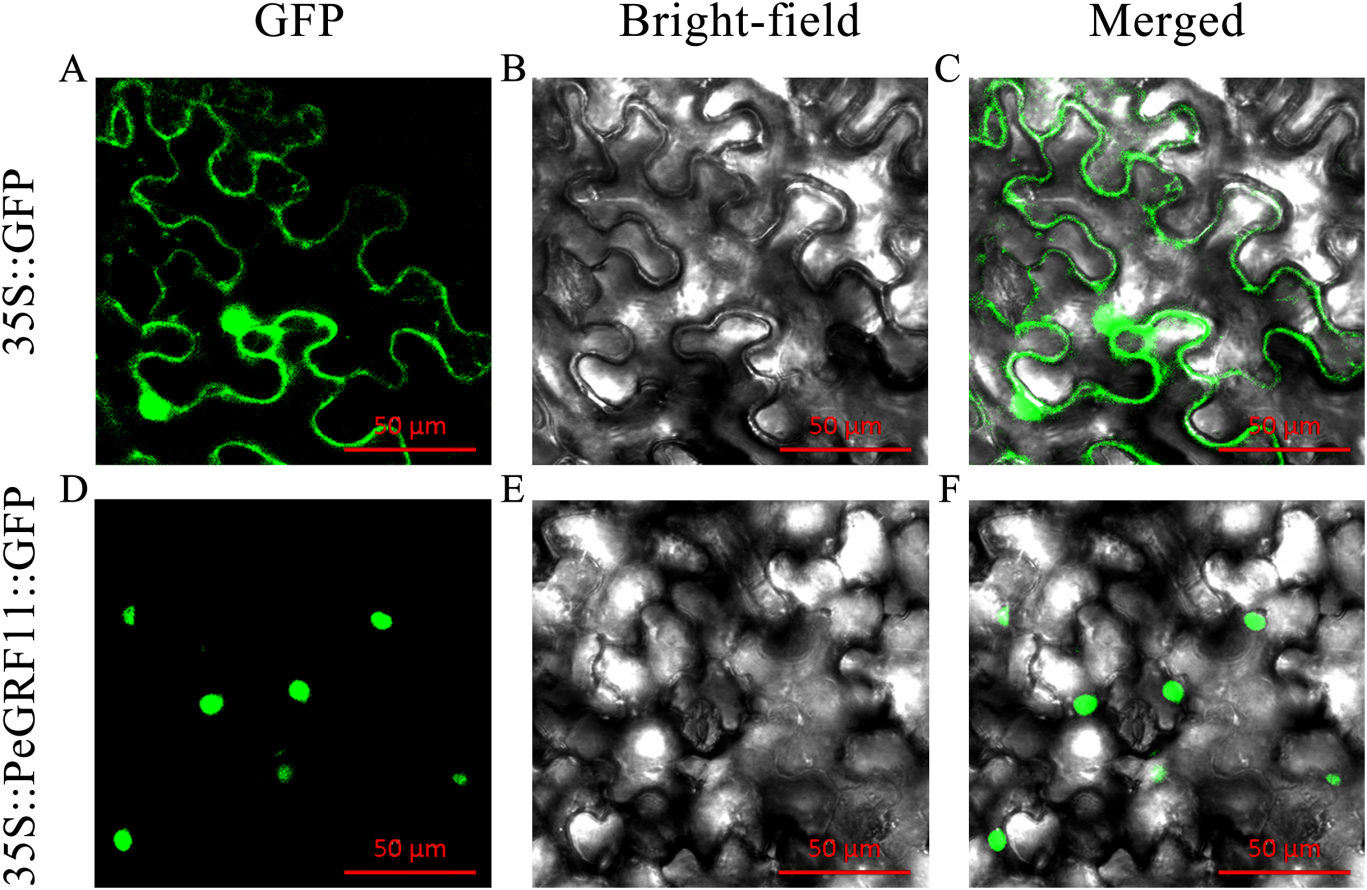
Subcellular localization of PeGRF11 in tobacco leaves. (A–C) The control and (D–F) 35S::PeGRF11::GFP fusion protein were independently expressed in tobacco leaf cells and observed using a fluorescence microscope. The green fluorescence channel and the bright field were used to construct the corresponding merged pictures. Scale bars = 50 μm.

Most TFs localize to the nucleus and play important roles in regulating target genes. PeGRF11 exhibited high expression levels in moso bamboo leaves and was significantly down-regulated after hormone treatment. The full-length CDS of PeGRF11 was amplified by specific primers (Table S1) and cloned into the pCAMBIA1305-GFP vector. Then, *Agrobacterium tumefaciens* independently harboring 35S::PeGRF11::GFP and the 35S::GFP control vector were infiltrated into *N. benthamiana* leaves. The 35S::PeGRF11::GFP fusion proteins were localized in the nucleus by GFP signals, while the expression of 35S::GFP alone was detected throughout the whole cell (Fig. 12).

### Transcriptional activation of PeGRF11

**Figure 13:**
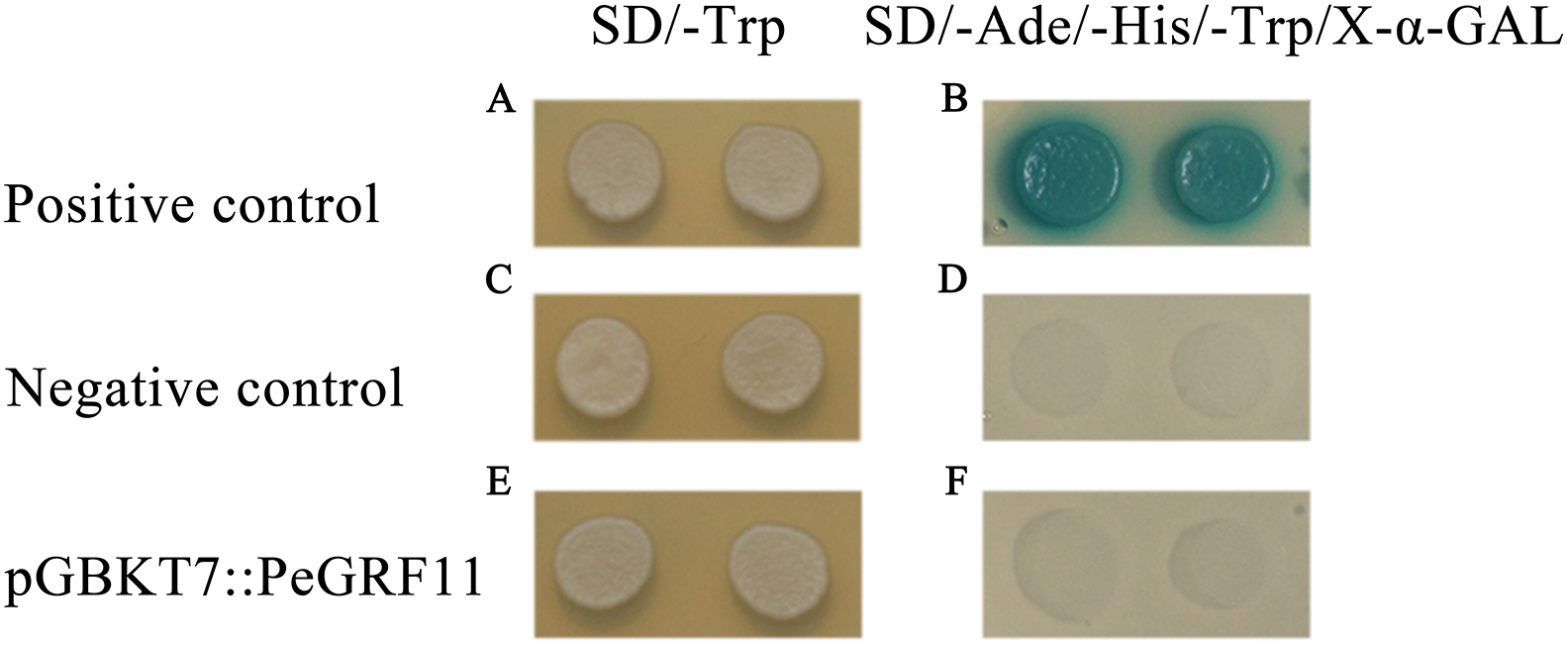
Transactivational analyses of the PeGRF11 protein in yeast. (A–F) The control vectors and fusion constructs were separately transformed into yeast strain Y2HGold and inoculated onto SD/−Trp and SD/−His/−Ade/−Trp/X-α-GAL plates.

To research the transcriptional activity of PeGRF11, the constructed pGBKT7::PeGRF11, positive control pGBKT7-53+pGADT7-T and empty pGBKT7 vectors were independently transformed into yeast strain Y2HGold, and these strains were inoculated onto SD/−Trp and SD/−Trp/−His/−Ade/X-α-GAL media, respectively. The three transformants containing pGBKT7::PeGRF11, pGBKT7-53+pGADT7-T (positive control) and pGBKT7 (negative control) vectors grew well on the SD/−Trp medium. On the SD/−Trp/−His/−Ade/X-α-GAL medium, the positive control group (pGBKT7-53+pGADT7-T) exhibited visible blue colonies with satisfactory growth states, while the pGBKT7::PeGRF11 and negative control transformants were unable to survive (Fig. 13). Thus, PeGRF11 did not possess transcriptional activity in yeast strains.

### PeGRF11 interactions with PeGIF3 as cotranscription factors

**Figure 14:**
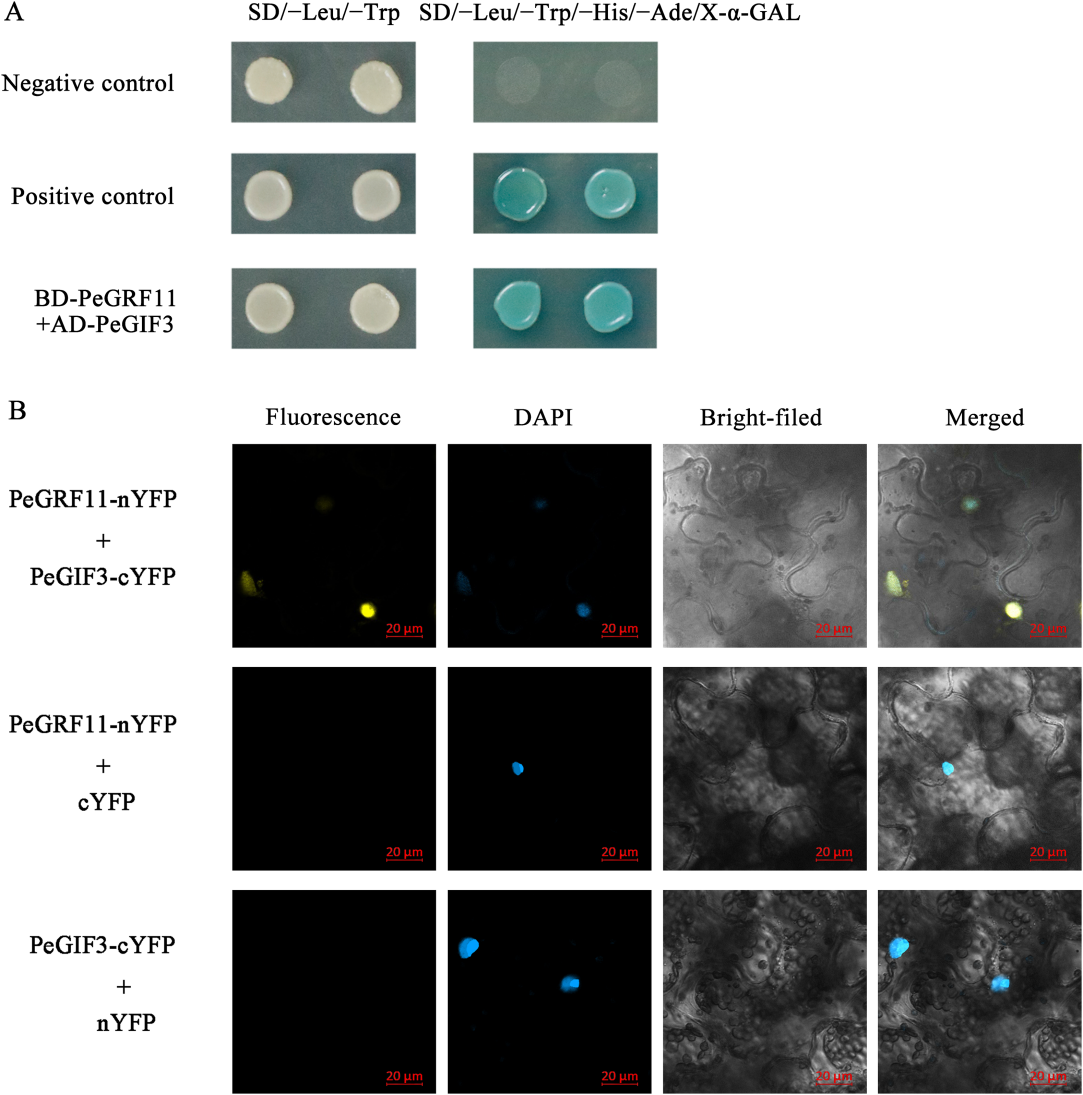
PeGRF11 interactions with PeGIF3 in yeast and in plant cells. (A) Yeast two-hybrid assay. Recombinant plasmids of BD-PeGRF11 and AD-PeGIF3 were cotransformed into yeast strain AH109 and then plated on a selective medium. (B) Fluorescence observed from the complementation of PeGRF11-nYFP and PeGIF3-cYFP, and colocalized with 4,6-diamidino-2-phenylindole stains in the nuclei of tobacco leaf cells. Scale bar = 20 μm.

Growth-regulating factor proteins interact with GIFs through the QLQ domain, and the GRF–GIF complex regulates leaf growth and morphology (Wu, Wang & Zhuang, 2017). To gain insights into the regulatory mechanisms of the *GRF* genes in moso bamboo, we performed yeast two-hybrid and BiFC assays. As depicted in Fig. 14A, BD-PeGRF11 and AD-PeGIF3 co-transformed yeast (AH109) cells grew well and turned blue on SD/−Leu/−Trp/−His/−Ade/X-α-GAL selective medium, similar to the positive control (pGBKT7-53+pGADT7-T), suggesting that PeGRF11 interacted with PeGIF3 in yeast. Furthermore, the constructed PeGRF11-N-YFP and PeGIF3-C-YFP vectors were co-transformed into tobacco (*N. benthamiana*) leaves for the BiFC assays. YFP signals were clearly distributed in the nuclear compartment based on staining with 4,6-diamidino-2-phenylindole (Fig. 14B). In contrast, YFP signals were not detected in the two negative controls (PeGRF11-N-YFP co-expressed with unfused C-YFP and PeGIF3-C-YFP co-expressed with unfused N-YFP). Thus, PeGRF11 physically interacted with PeGIF3 in yeast and in planta.

## Discussion

### GRFs in moso bamboo

As TFs regulating plant growth and development, GRFs have been studied since the discovery of *OsGRF1* (Omidbakhshfard et al., 2015; Van der Knaap, Kim & Kende, 2000). The genome-wide identification of *GRF* gene family has been reported in various plant species, including rice (Choi, Kim & Kende, 2004), maize (Zhang et al., 2008), *Arabidopsis* (Kim, Choi & Kende, 2003), Chinese cabbage (Wang et al., 2014), Chinese pear (Cao et al., 2016a) and tomato (Khatun et al., 2017). Nevertheless, little was known of the *GRF* gene family in moso bamboo. Here, 18 *GRF* genes containing the highly conserved QLQ and WRC domains in their N-terminal regions were identified from moso bamboo genome (Table 1; Fig. 1). The 54 *GRF* genes from rice, maize, *Brachypodium distachyon* and moso bamboo clustered into three major subfamilies based on the phylogenetic analysis, with moso bamboo *GRF* genes distributed across all three subfamilies (Fig. 2). Moso bamboo GRF proteins clustered onto relatively short branches with rice or *Brachypodium distachyon*, comparing with maize, which indicated that the evolutionary relationships between the moso bamboo GRF family in and those of rice and *Brachypodium* were closer than with the GRF family of maize (Peng et al., 2013). Thus, the *GRF* genes, which might share a common ancestor, had gone in distinct evolutionary directions among the different lineages. Furthermore, motif compositions and exon/intron structures among members of the same subfamily possessed certain similar features. In contrast, *GRF* genes from different subfamilies did not share consistent features. These results were in accordance with previous studies in tobacco (Zhang et al., 2018) and Chinese pear (Cao et al., 2016a), which further supported the close evolutionary relationships within GRF subfamilies and the dependability of our phylogenetic analysis. Additionally, the similar characteristics within the subfamilies might indicate that the moso bamboo GRF members had similar functions in plant growth and development.

### Molecular characterization of GRFs

The QLQ domain, a protein-binding region, could combine with GIFs to form a complex involving in the regulation of plant growth and development. AtGRF1 interacted with AtGIF1 to regulate the growth and shape of leaves and petals (Kim & Kende, 2004). The interplay between maize GRF10 and GIF2 formed a complex that might participate in the plant morphology formation process (Wu et al., 2014). PeGRF11, a homolog of ZmGRF10, was cloned, and we isolated PeGIF3, which was high consistency with ZmGIF2, to investigate this hypothesis in moso bamboo (Fig. S6). The yeast two-hybridization and BiFC assays revealed that the PeGRF11 and PeGIF3 proteins interacted with each other to form a complex that might be involved in the regulatory network of moso bamboo (Fig. 14).

The WRC domain served as a nuclear localization signal (Omidbakhshfard et al., 2015). Nuclear localization signals had been confirmed for several GRF members, such as OsGRF4 and OsGRF10 (Kuijt et al., 2014; Sun et al., 2016). In GO annotation analysis, the nuclear-related components were abundant in the cellular component category, which was related to the functional domain of the GRF transcription factor. PeGRF11 contained a WRC domain in its N-terminal region (Fig. 1), and its fusion protein was visibly expressed in the nucleus, which further verified this feature (Fig. 12).

Additionally, the C-terminal regions acted as transactivation domains, as shown by the transactivation activity assay of *AtGRF1* and *AtGRF2* fragments (Kim & Kende, 2004). *ZmGRF10*, defined as endogenous truncated gene owing to the loss of the entire C-terminal region, showed no transcriptional activity in yeast (Wu et al., 2014). Similarly, *PeGRF11* was not transcriptionally activated in yeast probably owing to the lack of a C-terminal domain (Fig. 13; Fig. S6). Thus, we hypothesized that the loss of the C-terminal region of PeGRF11 might occur during a gene duplication event.

### Potential functions of GRFs in moso bamboo

The expression profiles of *PeGRF* gene in different tissues provided valuable clues for predicting gene functions during plant growth and development. GO annotation analysis indicated that the moso bamboo GRF genes involved in different biological processes, including leaf development (GO:0048366), seed development (GO:0048316), developmental process (GO:0032502), etc. These suggested that the moso bamboo GRF genes might have functional differentiation. *GRF* genes showed high expression levels in relatively actively growing tissues, such as germinating seeds and buds (Horiguchi, Kim & Tsukaya, 2005; Kim, Choi & Kende, 2003; Kim et al., 2012; Zhang et al., 2018). Based on a microarray data analysis, PeGRF genes were expressed more highly in moso bamboo leaf and early panicle where cell proliferation vigorously occurred in. Similar phenomenon occurred in tobacco (Zhang et al., 2018) and *Brassica napus* (Ma et al., 2017). *Arabidopsis* overexpressing *AtGRF5* exhibited larger leaves than normal and could enhance cell proliferation in leaf primordia (Horiguchi, Kim & Tsukaya, 2005). However, the overexpression of *ZmGRF10* resulted in smaller leaf size and shorter plant heights because of the decrease in cell proliferation (Wu et al., 2014). Therefore, GRFs might regulate leaf size by enhancing or decreasing cell proliferation. Furthermore, *PeGRF11*, a homolog of *ZmGRF10* in moso bamboo, was highly expressed in leaves, which might affect biological pathways by involving in plant growth and development.

Plant hormones, which are metabolic products in plants, are involved in plant growth and development. GRFs played roles in responding to plant hormones (Khatun et al., 2017; Van der Knaap, Kim & Kende, 2000). Seven *OsGRFs* were strongly up-regulated owing to the application of GA3 (Choi, Kim & Kende, 2004). However, *AtGRF* gene expression levels did not remarkably change after GA3 treatments (Kim, Choi & Kende, 2003). *NtabGRF* genes significantly respond to various hormones, including GA3, ABA, IAA, BR and 6-BA (Zhang et al., 2018). Most *Camellia sinensis GRF* genes were up-regulated or maintained when treated with salicylic acid or IAA, whereas only one gene was up-regulated after a GA3 treatment (Wu, Wang & Zhuang, 2017). Here, the expression levels of nine *PeGRF* genes showed remarkable changes after GA3 treatments; six members were significantly up-regulated to fourfold or more compared with at 0 h, and three members were significantly down-regulated to less than 0.5-fold (Fig. 9). Similarly, *PeGRF* genes had significant responses to MeJA and ABA treatments (Figs. 10 and 11). Thus, moso bamboo GRF members might be necessary for regulatory hormone feedback mechanisms. Promoter analysis also provided important information on potential functions and gene expression changes in response to hormone treatments. GARE and P-box motifs, which were GA-responsive elements, were identified in the promoter regions of 10 *PeGRF*s. MeJA-responsiveness elements (including CGTCA and TGACG motifs) were positioned in the promoter regions of moso bamboo *GRF* genes. In addition, several *PeGRF*s contained ABRE, IIb and CE1 motifs, which were involved in the ABA responses during the growth process. Therefore, we hypothesized that *GRFs* might have different roles in the regulation hormonal feedback mechanisms and physiological processes.

## Conclusions

In this study, we identified 18 *PeGRF* genes from moso bamboo genome, which were further classified into three subfamilies based on phylogenetic relationship analysis. Analyses of motif compositions and exon/intron structures showed that the most members had conserved structural features within the same subfamily. The Ka/Ks ratios of the paralogous (*Pe*-*Pe*) and orthologous (*Pe*-*Os*, *Pe*-*Bd*) gene pairs were less than 1, indicating that the GRF families of these species had undergone strong purifying selection. We also performed the qRT-PCR analysis of moso bamboo *GRF* genes under hormone (GA3, MeJA and ABA) treatments, which displayed that the *PeGRF* gene had significant responses to hormone treatment. Besides, promoter analysis of *PeGRFs* also suggested that they might be necessary for regulatory hormone feedback mechanisms. The GO annotation analysis showed biological process for the PeGRF regulation in plant development. The expression profiles of moso bamboo *GRFs* were explored by transcriptome data, from which we screened PeGRF11 which had high expression levels in various tissues, and performed subcellular localization and transactivation activity, yeast two-hybridization and BiFC assays. The results revealed valuable clues to the functions of PeGRFs in moso bamboo growth and development.

## Supplemental Information

10.7717/peerj.7510/supp-1 Supplemental Information 1 Primers used in this study.

10.7717/peerj.7510/supp-2 Supplemental Information 2 Details of *GRF* genes from rice, maize and *Brachypodium distachyon*.

10.7717/peerj.7510/supp-3 Supplemental Information 3 Detailed information on 20 *PeGRF* motifs.

10.7717/peerj.7510/supp-4 Supplemental Information 4 Ka/Ks analysis and divergence times for orthologs pairs (*Pe-Os* and *Pe-Bd*).

10.7717/peerj.7510/supp-5 Supplemental Information 5 Gene ontology annotation of PeGRF proteins.

10.7717/peerj.7510/supp-6 Supplemental Information 6 Promoter analysis of the *PeGRF* gene family.

10.7717/peerj.7510/supp-7 Supplemental Information 7 Microarray data of 18 *GRF* genes in moso bamboo.

10.7717/peerj.7510/supp-8 Supplemental Information 8 Specific primers for amplifying 18 *PeGRF* genes using qRT-PCR.

10.7717/peerj.7510/supp-9 Supplemental Information 9 ML phylogenetic analysis of GRF from moso bamboo, rice, maize and *Brachypodium distachyon*.

10.7717/peerj.7510/supp-10 Supplemental Information 10 Phylogenetic tree of GRFs in moso bamboo. The tree was divided into subfamilies A, B and C.

10.7717/peerj.7510/supp-11 Supplemental Information 11 Sliding window analysis for the Ka/Ks ratio of the *Pe-Pe* gene pairs.

10.7717/peerj.7510/supp-12 Supplemental Information 12 Sliding window analysis for the Ka/Ks ratio of the *Pe-Os* orthologous gene pairs.

10.7717/peerj.7510/supp-13 Supplemental Information 13 Sliding window analysis for the Ka/Ks ratio of the *Pe-Bd* orthologous gene pairs.

10.7717/peerj.7510/supp-14 Supplemental Information 14 Alignment of the amino acid sequences of the homologous gene pairs *PeGRF11/ZmGRF10* and *PeGIF3/ZmGIF2*.

10.7717/peerj.7510/supp-15 Supplemental Information 15 Protein sequences of PeGRFs, OsGRFs, ZmGRFs and BdGRFs.

10.7717/peerj.7510/supp-16 Supplemental Information 16 Gene sequences of 18 PeGRFs.

10.7717/peerj.7510/supp-17 Supplemental Information 17 CDS sequences of 18 PeGRFs.

## Abbreviation

GRF: Growth-regulating factor
GIF: GRF–interacting factor
BiFC: bimolecular fluorescence complementation assays
NCBI: National Center of Biotechnology Information
GA3: Gibberellin 3
MeJA: Methyl Jasmonate
ABA: abscisic acid
Ks: number of synonymous substitutions per synonymous site
Ka: number of non-synonymous substitutions per non-synonymous site
CDS: coding sequence
qRT-PCR: quantitative real-time PCR.

## Additional Information and Declarations

### Data Availability

The following information was supplied regarding data availability: Moso Bamboo (Phyllostachys heterocycla var. pubescens) data is available at the National Center For Gene Research, accessible here: http://server.ncgr.ac.cn/bamboo/down.php. The bamboo CDS sequence and protein sequence data files are available in P_heterocycla_v1.0.genemodel-cds-DNA.gz and P_heterocycla_v1.0.genemodel-cds-PROTEIN.gz. After unzipping the files, search for accession numbers (“Gene identifier”): PH01000005G1660, PH01000045G1330, PH01000083G1570, PH01000152G0440, PH01000197G1460, PH01000387G0730, PH01000513G0530, PH01000842G0540, PH01001096G0260, PH01001291G0040, PH01001304G0330, PH01001605G0350, PH01002169G0420, PH01002618G0290, PH01002701G0020, PH01003592G0180, PH01005386G0020, PH01087379G0010.

Assembled scaffolds of moso bamboo genome are available in P_heterocycla_v1.0.Scaffolds.gz. We extracted the bamboo gene fasta file using the Perl program based on the location information (“Location coordinates”) of the PeGRF gene.
