# Supplementary material for: Genome-wide identification of growth-regulating factors in moso bamboo (Phyllostachys edulis): in silico and experimental analyses
Source: PeerJ. 2019 Sep 12;7:e7510. doi: 10.7717/peerj.7510 (PMC6769349; doi:10.7717/peerj.7510)
Supplement: Supplemental Information 2 [file peerj-07-7510-s002.docx]

**Table S2.** Details of *GRF* genes from rice, maize and *Brachypodium distachyon*

| Name | Gene Identifier | Name | Gene Identifier | Name | Gene Identifier |
| --- | --- | --- | --- | --- | --- |
|  |  |  |  |  |  |
| OsGRF1 | LOC_Os02g45570.1 | ZmGRF1 | EF515840 | BdGRF1 | Bradi1g09900.1 |
| OsGRF2 | LOC_Os02g47280.1 | ZmGRF2 | EF515841 | BdGRF2 | Bradi1g12650.3 |
| OsGRF3 | LOC_Os02g53690.1 | ZmGRF3 | EF515842 | BdGRF3 | Bradi2g14320.1 |
| OsGRF4 | LOC_Os03g47140.1 | ZmGRF4 | EF515843 | BdGRF4 | Bradi4g16450.1 |
| OsGRF5 | LOC_Os03g51970.1 | ZmGRF5 | EF515844 | BdGRF5 | Bradi5g20607.1 |
| OsGRF6 | LOC_Os04g48510.1 | ZmGRF6 | EF515845 | BdGRF6 | Bradi1g28400.1 |
| OsGRF7 | LOC_Os04g51190.1 | ZmGRF7 | EF515846 | BdGRF7 | Bradi1g46427.1 |
| OsGRF8 | LOC_Os06g02560.1 | ZmGRF8 | EF515847 | BdGRF8 | Bradi1g50597.1 |
| OsGRF9 | LOC_Os06g10310.1 | ZmGRF9 | EF515848 | BdGRF9 | Bradi3g51685.1 |
| OsGRF10 | LOC_Os07g28430.1 | ZmGRF10 | EF515849 | BdGRF10 | Bradi3g57267.1 |
| OsGRF11 | LOC_Os11g35030.1 | ZmGRF11 | EF515850 |  |  |
| OsGRF12 | LOC_Os12g29980.1 | ZmGRF12 | EF515851 |  |  |
|  |  | ZmGRF13 | EF515852 |  |  |
|  |  | ZmGRF14 | EF515853 |  |  |
