# Supplementary material for: Genome-wide identification of growth-regulating factors in moso bamboo (Phyllostachys edulis): in silico and experimental analyses
Source: PeerJ. 2019 Sep 12;7:e7510. doi: 10.7717/peerj.7510 (PMC6769349; doi:10.7717/peerj.7510)
Supplement: Supplemental Information 3 [file peerj-07-7510-s003.docx]

**Table S3.** Detailed information on 20 *PeGRF* motifs

| Motif | Width | Best Possible Match |
| --- | --- | --- |
| 1 | 47 | DPEPGRCRRTDGKKWRCSREAVPDSKYCERHMHRGRNRSRKPVESQT |
| 2 | 41 | RAPFTAAQWQELEHQALIYKYLAAGAPVPPDLVLPIRKSVA |
| 3 | 57 | ISLPKVEREPLSFFGTDFGTVDSVKQENQPLRPFFDEWPKARDSWSELADENSNLTS |
| 4 | 100 | KPGSVNYWTDSLNRTMLSKEKANKPAEDNNAPLLNSTNRQPTLSLJCQPKQQNKPDKFSPTVDSESISSNTILKPWESSNQQSNKNISCTRLHDRECLQS |
| 5 | 100 | ENVNLLGSQSLNEHQSAVFLQHFVDWPRTPAQGALGWPDAEEMQAQRTQLSISAPMASSDLSSASTSPIHEKLMLSPLKLRREYSPIGLSFAANKDEVCQ |
| 6 | 57 | KYAAYGIRSLADEHSQLITEAIDTPIDNSWRLLPSQVPSFPLSSYRQLGALSDLGQN |
| 7 | 21 | AQKYPTLMGLATLCLDFGKNP |
| 8 | 19 | FSATQLSISIPMASSDFSV |
| 9 | 15 | FYHHPALGYGSYFGK |
| 10 | 54 | PKESDLGLMKRSNFTQAASYPSPFLDEQKILRFSKAARTLPSGMDFGRPNEHMF |
| 11 | 41 | VNQRNTGSLFPFSKQHNPFDVTSSRPDFGLVSSDSLMSSPH |
| 12 | 21 | KATEGGKKTDDKSSSSKKLAV |
| 13 | 56 | MAEEKEADSPQPPSKLPRLSCADPNAGAVTMADSSPLVLGLGLGLGGDSCGERGVE |
| 14 | 9 | GWGSFQLGY |
| 15 | 12 | NRMSWRPLYQGF |
| 16 | 16 | YENVVRKALERKAHCR |
| 17 | 18 | QQPVKSYAANVTDPFPRQ |
| 18 | 29 | RKTAKEIPAAGSLSSPVSQGSFKKAKVNE |
| 19 | 11 | MGMGGYQQQQQ |
| 20 | 16 | QLHMDNAAPYAAVGGG |
