# Supplementary material for: Genome-wide identification of growth-regulating factors in moso bamboo (Phyllostachys edulis): in silico and experimental analyses
Source: PeerJ. 2019 Sep 12;7:e7510. doi: 10.7717/peerj.7510 (PMC6769349; doi:10.7717/peerj.7510)
Supplement: Supplemental Information 4 [file peerj-07-7510-s004.docx]

Table S4. Ka/Ks analysis and divergence times for orthologs pairs (Pe-Os and Pe-Bd)

| Pe-Os | Ka | Ks | Ka/Ks | Duplication Date (MY) |
| --- | --- | --- | --- | --- |
| PeGRF17/OsGRF9 | 0.11613 | 0.38182 | 0.304 | 29.37076923 |
| PeGRF16/OsGRF9 | 0.10325 | 0.3738 | 0.276 | 28.75384615 |
| PeGRF1/OsGRF8 | 0.15232 | 0.31966 | 0.476 | 24.58923077 |
| PeGRF12/OsGRF7 | 0.3101 | 0.61431 | 0.505 | 47.25461538 |
| PeGRF6/OsGRF6 | 0.06306 | 0.34644 | 0.182 | 26.64923077 |
| PeGRF9/OsGRF6 | 0.06919 | 0.36541 | 0.189 | 28.10846154 |
| PeGRF3/OsGRF5 | 0.06682 | 0.40362 | 0.166 | 31.04769231 |
| PeGRF2/OsGRF5 | 0.06936 | 0.3495 | 0.198 | 26.88461538 |
| PeGRF5/OsGRF4 | 0.08548 | 0.38771 | 0.22 | 29.82384615 |
| PeGRF14/OsGRF4 | 0.21595 | 0.47346 | 0.456 | 36.42 |
| PeGRF4/OsGRF4 | 0.21701 | 0.63528 | 0.342 | 48.86769231 |
| PeGRF4/OsGRF3 | 0.12345 | 0.21168 | 0.583 | 16.28307692 |
| PeGRF5/OsGRF3 | 0.23315 | 0.508 | 0.459 | 39.07692308 |
| PeGRF14/OsGRF3 | 0.33903 | 0.59111 | 0.574 | 45.47 |
| PeGRF13/OsGRF12 | 0.17841 | 0.40022 | 0.446 | 30.78615385 |
| PeGRF8/OsGRF12 | 0.19492 | 0.39579 | 0.492 | 30.44538462 |
| PeGRF15/OsGRF11 | 0.09192 | 0.53512 | 0.172 | 41.16307692 |
| PeGRF11/OsGRF11 | 0.01836 | 0.51184 | 0.036 | 39.37230769 |
| PeGRF7/OsGRF11 | 0.28001 | 0.60958 | 0.459 | 46.89076923 |
| PeGRF13/OsGRF10 | 0.09852 | 0.19023 | 0.518 | 14.63307692 |
| PeGRF8/OsGRF10 | 0.08696 | 0.23774 | 0.366 | 18.28769231 |
| PeGRF18/OsGRF1 | 0.09188 | 0.28705 | 0.32 | 22.08076923 |

| Pe-Bd | Ka | Ks | Ka/Ks | Duplication Date (MY) |
| --- | --- | --- | --- | --- |
| PeGRF13/BdGRF9 | 0.17012 | 0.2299 | 0.74 | 17.68461538 |
| PeGRF8/BdGRF9 | 0.14842 | 0.22328 | 0.665 | 17.17538462 |
| PeGRF3/BdGRF8 | 0.07647 | 0.26603 | 0.287 | 20.46384615 |
| PeGRF2/BdGRF8 | 0.0919 | 0.29943 | 0.307 | 23.03307692 |
| PeGRF10/BdGRF7 | 0.32039 | 0.29361 | 1.091 | 22.58538462 |
| PeGRF15/BdGRF6 | 0.12885 | 0.30667 | 0.42 | 23.59 |
| PeGRF11/BdGRF6 | 0.06288 | 0.33617 | 0.187 | 25.85923077 |
| PeGRF7/BdGRF6 | 0.34043 | 0.60152 | 0.566 | 46.27076923 |
| PeGRF4/BdGRF5 | 0.19343 | 0.28633 | 0.676 | 22.02538462 |
| PeGRF5/BdGRF5 | 0.22589 | 0.48469 | 0.466 | 37.28384615 |
| PeGRF14/BdGRF5 | 0.29498 | 0.53078 | 0.556 | 40.82923077 |
| PeGRF1/BdGRF4 | 0.12772 | 0.29686 | 0.43 | 22.83538462 |
| PeGRF15/BdGRF3 | 0.16164 | 0.37229 | 0.434 | 28.63769231 |
| PeGRF11/BdGRF3 | 0.04444 | 0.33299 | 0.133 | 25.61461538 |
| PeGRF7/BdGRF3 | 0.27365 | 0.60339 | 0.454 | 46.41461538 |
| PeGRF16/BdGRF2 | 0.09607 | 0.37652 | 0.255 | 28.96307692 |
| PeGRF17/BdGRF2 | 0.0988 | 0.34631 | 0.285 | 26.63923077 |
| PeGRF18/BdGRF10 | 0.2464 | 0.36088 | 0.683 | 27.76 |
| PeGRF9/BdGRF1 | 0.09302 | 0.35953 | 0.259 | 27.65615385 |
| PeGRF6/BdGRF1 | 0.08756 | 0.3651 | 0.24 | 28.08461538 |
