# Supplementary material for: Genome-wide identification of growth-regulating factors in moso bamboo (Phyllostachys edulis): in silico and experimental analyses
Source: PeerJ. 2019 Sep 12;7:e7510. doi: 10.7717/peerj.7510 (PMC6769349; doi:10.7717/peerj.7510)
Supplement: Supplemental Information 7 [file peerj-07-7510-s007.docx]

**Table S7.** Microarray data of 18 *GRF* genes in moso bamboo

| name | leaf | panicle1 | panicle2 | root | rhizome |
| --- | --- | --- | --- | --- | --- |
| *PeGRF1* | 13.0632 | 9.82538 | 6.7292 | 8.60749 | 6.9694 |
| *PeGRF2* | 4.47738 | 3.17077 | 3.03166 | 10.9903 | 7.79373 |
| *PeGRF3* | 8.01156 | 4.98883 | 5.04484 | 14.2438 | 11.5029 |
| *PeGRF4* | 3.50562 | 5.7157 | 9.23149 | 0.124737 | 0.339232 |
| *PeGRF5* | 1.35796 | 1.50119 | 1.35113 | 0.0419146 | 0 |
| *PeGRF6* | 3.01747 | 4.70852 | 6.11404 | 28.3868 | 30.544 |
| *PeGRF7* | 5.15638 | 4.93158 | 3.00562 | 3.29593 | 3.06218 |
| *PeGRF8* | 4.02087 | 1.90901 | 1.07156 | 6.05537 | 2.97157 |
| *PeGRF9* | 3.47044 | 3.30037 | 1.65625 | 10.6551 | 13.2553 |
| *PeGRF10* | 1.16161 | 0.94044 | 0.810866 | 3.13325 | 2.7096 |
| *PeGRF11* | 16.345 | 20.3438 | 18.7237 | 11.2642 | 10.493 |
| *PeGRF12* | 1.23306 | 1.20466 | 1.86838 | 2.0872 | 2.1228 |
| *PeGRF13* | 0.925968 | 1.78063 | 0.732317 | 2.75823 | 1.99971 |
| *PeGRF14* | 1.35945 | 1.1656 | 0.260458 | 0.179278 | 0.227463 |
| *PeGRF15* | 6.91525 | 7.25185 | 9.66764 | 4.13395 | 4.14023 |
| *PeGRF16* | 1.2341 | 2.80275 | 1.16417 | 0.765283 | 0.979388 |
| *PeGRF17* | 3.69862 | 2.86301 | 3.95053 | 0.512414 | 0.503288 |
| *PeGRF18* | 3.17018 | 2.53423 | 2.04595 | 9.25665 | 5.60216 |
