# Supplementary material for: Genome-wide identification of growth-regulating factors in moso bamboo (Phyllostachys edulis): in silico and experimental analyses
Source: PeerJ. 2019 Sep 12;7:e7510. doi: 10.7717/peerj.7510 (PMC6769349; doi:10.7717/peerj.7510)
Supplement: Supplemental Information 8 [file peerj-07-7510-s008.docx]

| **Gene name** |  | **Primer sequence**s |
| --- | --- | --- |
| *PeGRF1F* | Forward | GCATGGAGTAGGCAGTTCATCG |
|  | Reverse | AGAGGTCCGTCGTCAAGTGTTT |
| *PeGRF2F* | Forward | ACCTTGAGCCATCACAGGAACT |
|  | Reverse | GACCGCTCGTCGTCCATCT |
| *PeGRF3F* | Forward | CCGTTCAAGAAAGCCTGTGG |
|  | Reverse | AATGAACCTGCGACGAGCC |
| *PeGRF4F* | Forward | CTTGGGTACGGGTCCTACTTCG |
|  | Reverse | GCGTTTCCACAGGCTTTCTTG |
| *PeGRF5F* | Forward | CCCCTTTCGAGCTACCGTCA |
|  | Reverse | CGTCTGGTTCTCCTGCTTCACA |
| *PeGRF6F* | Forward | GCCTGCTCCTCCCCATCA |
|  | Reverse | CCATTTCTTGCCGTCCGTC |
| *PeGRF7F* | Forward | GGAGGAAATGGGAACGCTTGT |
|  | Reverse | CGAGATGGGTAGGCACTGGTAT |
| *PeGRF8F* | Forward | CGTCGCTGCCTCGTCCTT |
|  | Reverse | CACCTCCACTTCTTGCCATCA |
| *PeGRF9F* | Forward | GCAATGGCATCCTCTGACCTA |
|  | Reverse | TTCTCCCTGGCAAACTTCATCT |
| *PeGRF10F* | Forward | CAGCTACGGCCACCAACACC |
|  | Reverse | GCTCGGGAAGAGGGGAGAAGT |
| *PeGRF11F* | Forward | GCGGTGCCGACGAACAGA |
|  | Reverse | CTTGCCAGACGCAGATTTTGA |
| *PeGRF12F* | Forward | TCTGGGAGTGCTGACCCTGAG |
|  | Reverse | GCGGCCCCGATTTATGTG |
| *PeGRF13F* | Forward | TGCTCATCTACCGCTACTTCGC |
|  | Reverse | GGCTCCCCAGTCCCATCA |
| *PeGRF14F* | Forward | TGTGAAGCATGAGAACCAAACG |
|  | Reverse | TGGCAACGGAGAAGTCAGAGG |
| *PeGRF15F* | Forward | GAAGCCGCAGCATCAGCA |
|  | Reverse | TGGGTAGGCACAGGAACACG |
| *PeGRF16F* | Forward | CCCGCTGCTGGCTCATTG |
|  | Reverse | CGTGGGTTGTCGATTCGTAGAA |
| *PeGRF17F* | Forward | TGAGCGGCACATCAATAGGAA |
|  | Reverse | AATGAGCCAGCAGCGGGTA |
| *PeGRF18F* | Forward | GCAGGTGAGCCCGTTCCA |
|  | Reverse | GCTGTGCCCGTAGGCGTAGT |
| *TIP41* | Forward | AAAATCATTGTAGGCCATTGTCG |
|  | Reverse | ACTAAATTAAGCCAGCGGGAGTG |

**Table S8.**Specific primers for amplifying 18 *PeGRF* genes using qRT-PCR
