# Supplementary figures and images for: Genome-wide identification of growth-regulating factors in moso bamboo (Phyllostachys edulis): in silico and experimental analyses
Source: PeerJ. 2019 Sep 12;7:e7510. doi: 10.7717/peerj.7510 (PMC6769349; doi:10.7717/peerj.7510)

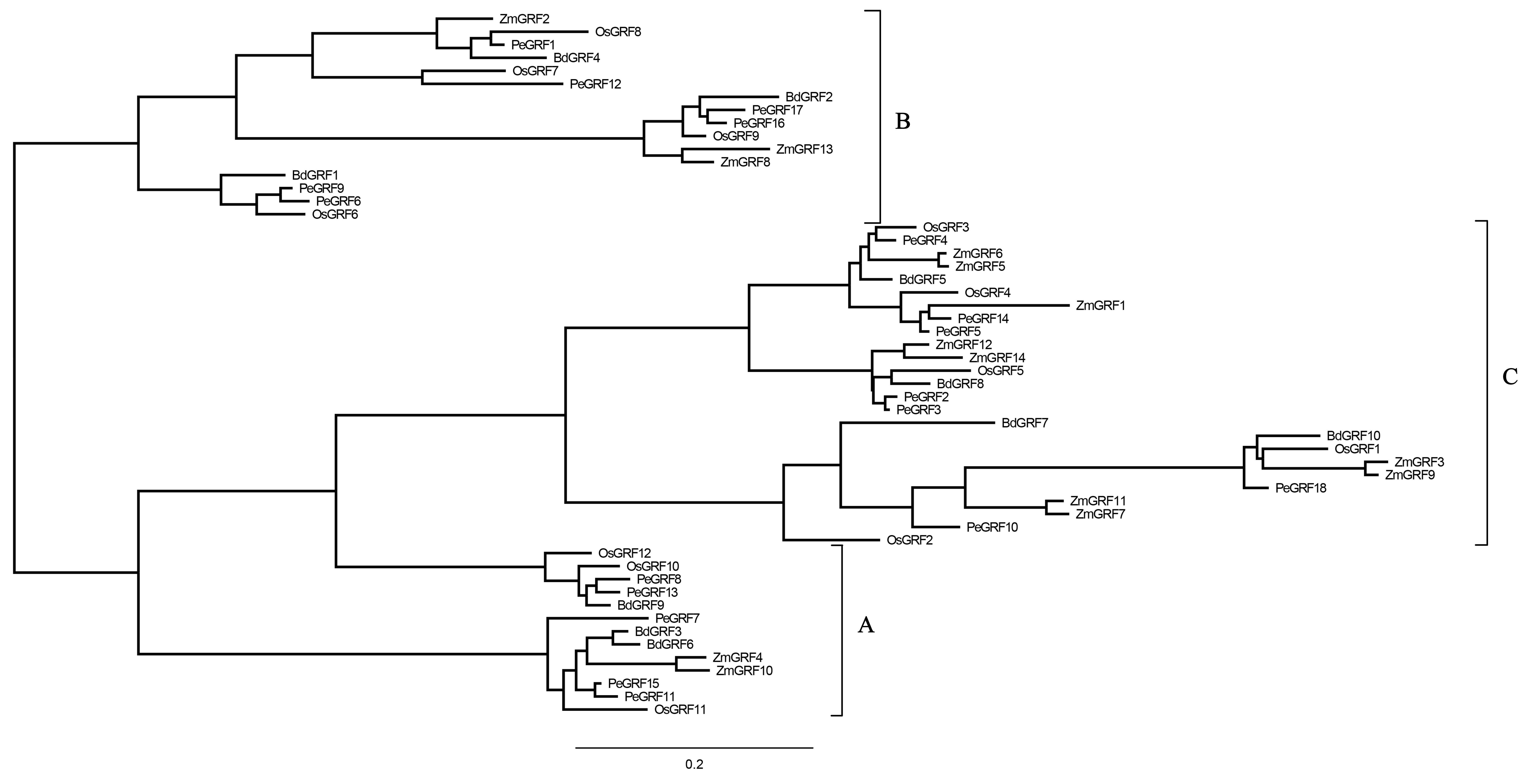

Supplement: Supplemental Information 9 [file peerj-07-7510-s009.png]

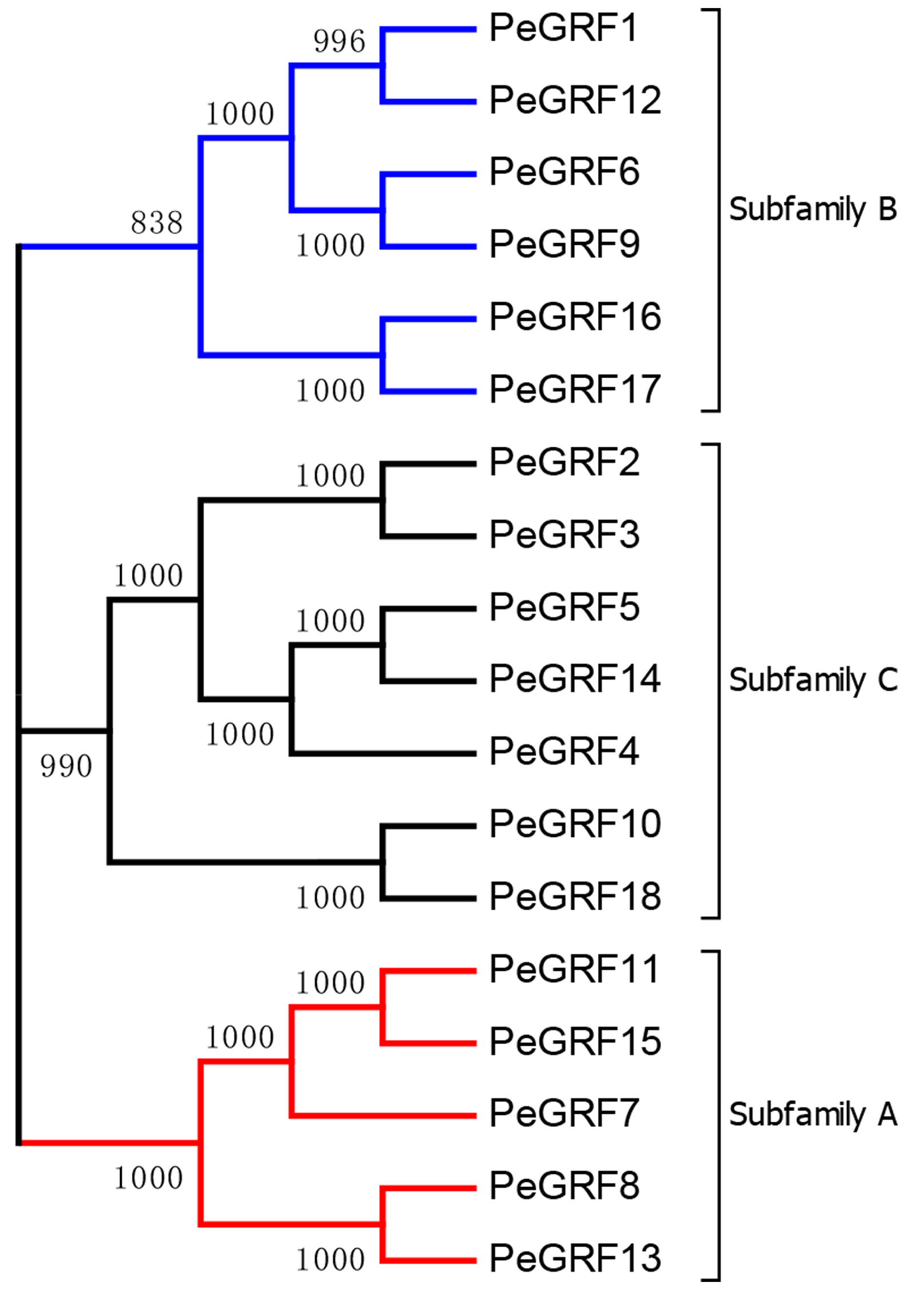

Supplement: Supplemental Information 10 [file peerj-07-7510-s010.png]

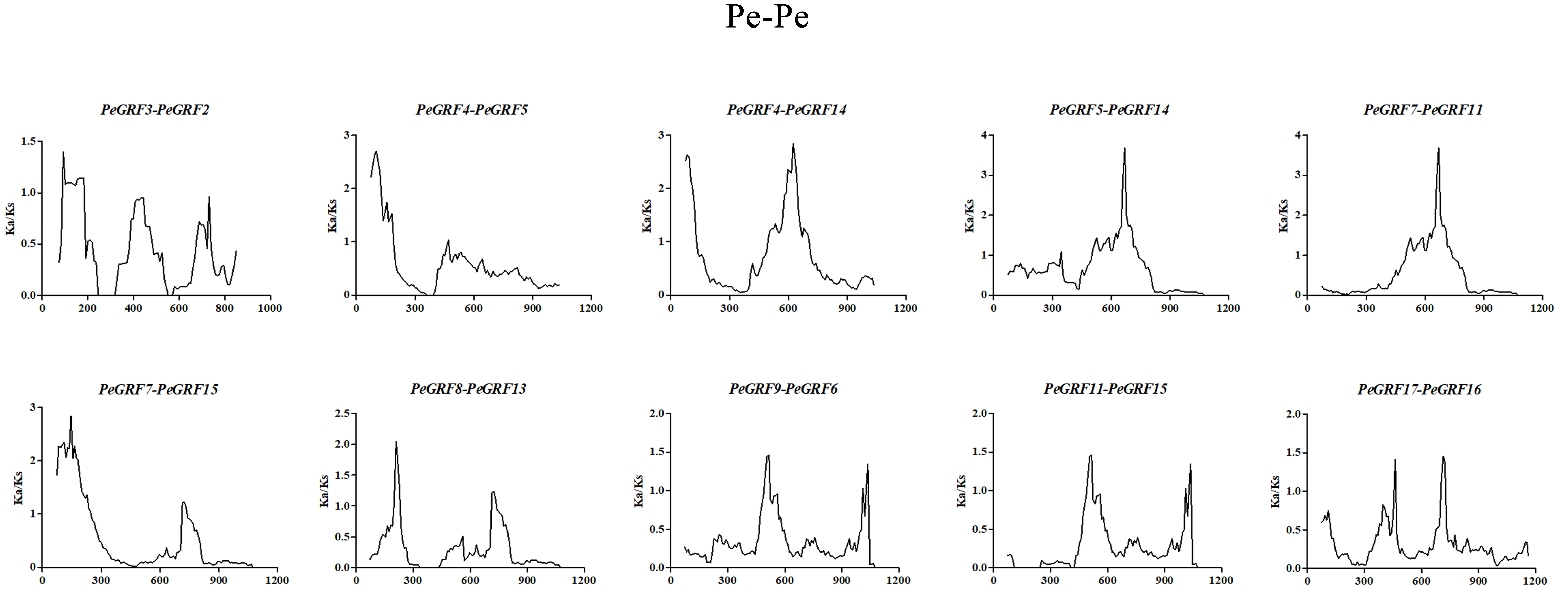

Supplement: Supplemental Information 11 [file peerj-07-7510-s011.png]

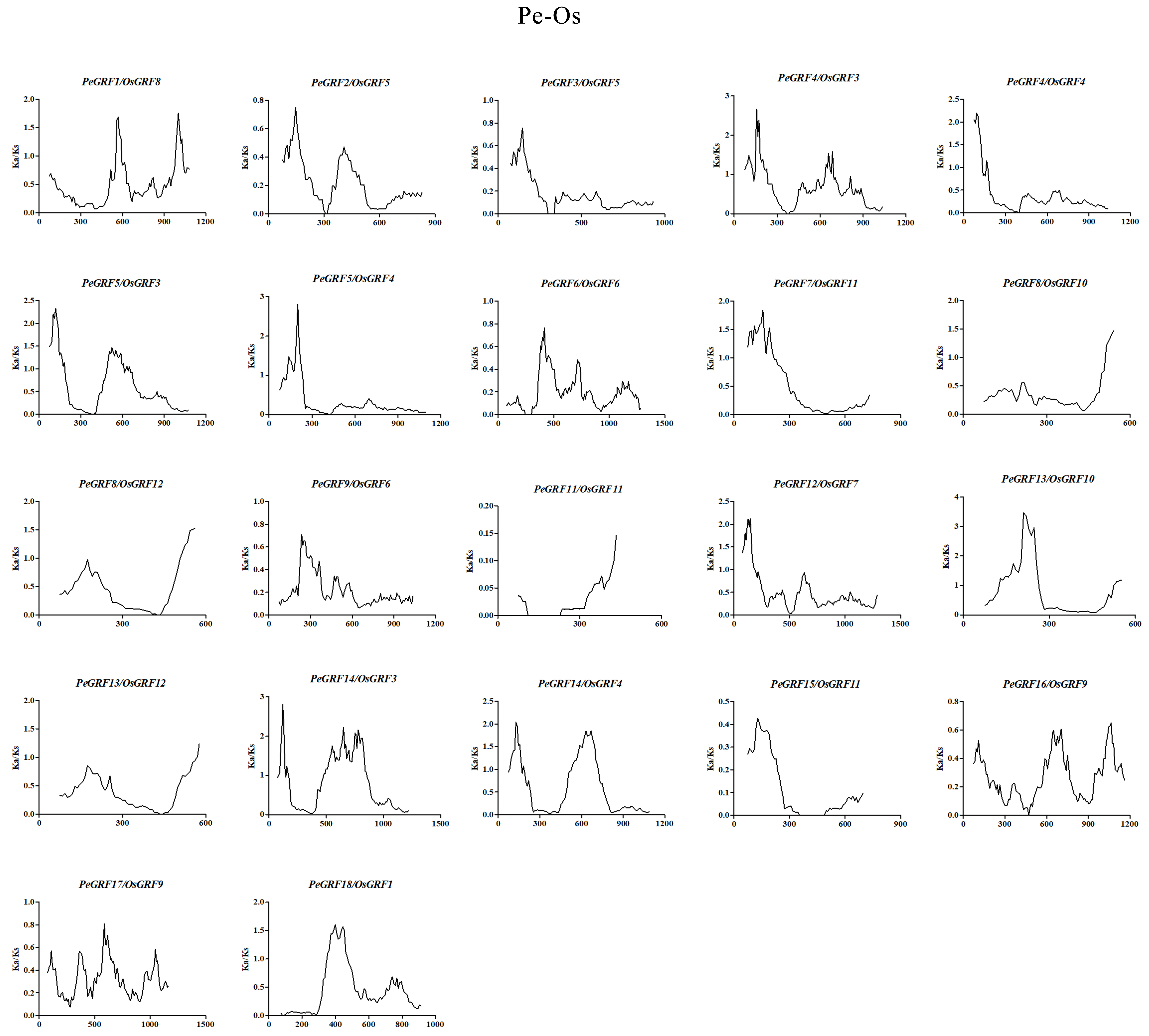

Supplement: Supplemental Information 12 [file peerj-07-7510-s012.png]

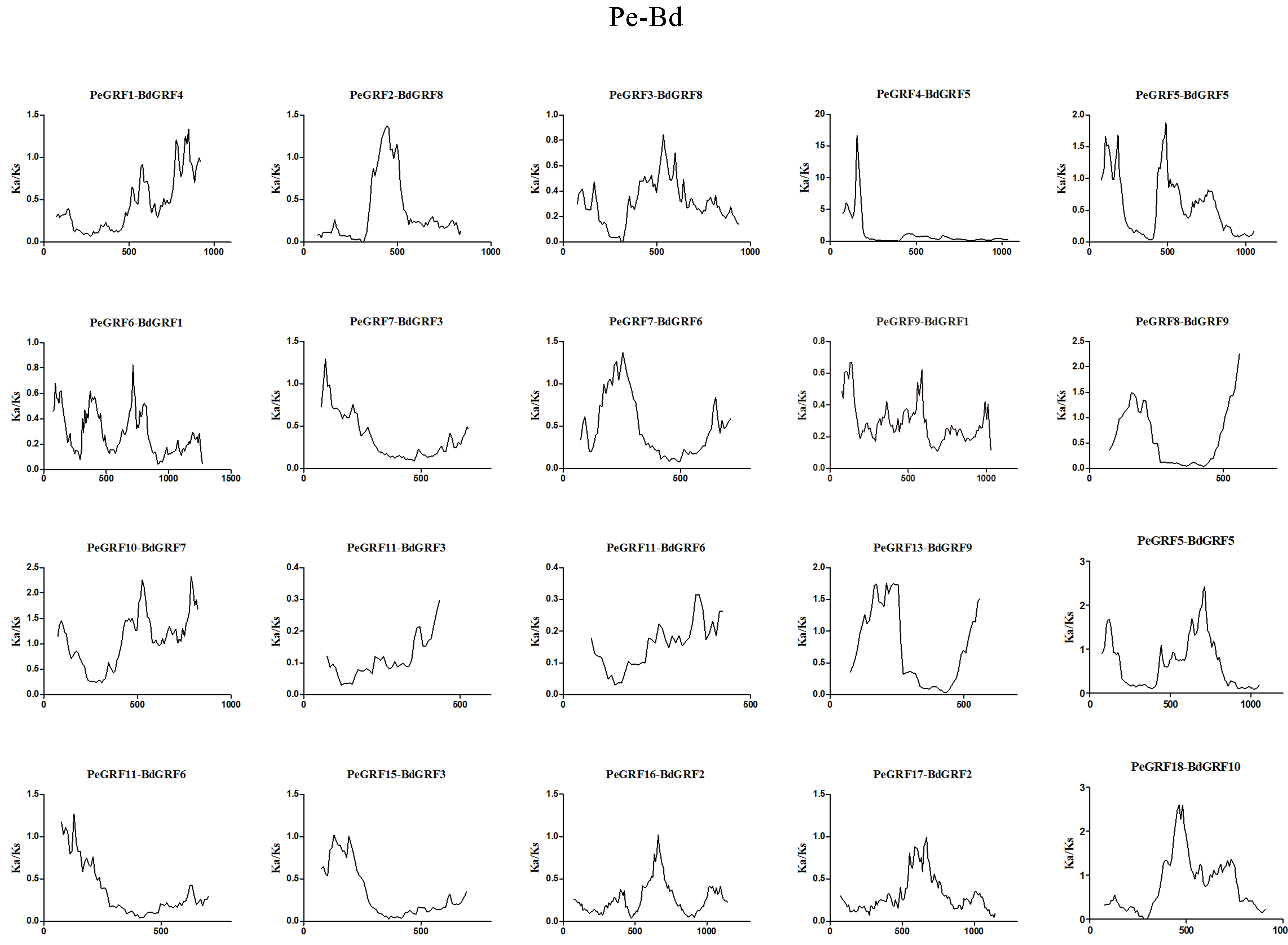

Supplement: Supplemental Information 13 [file peerj-07-7510-s013.png]

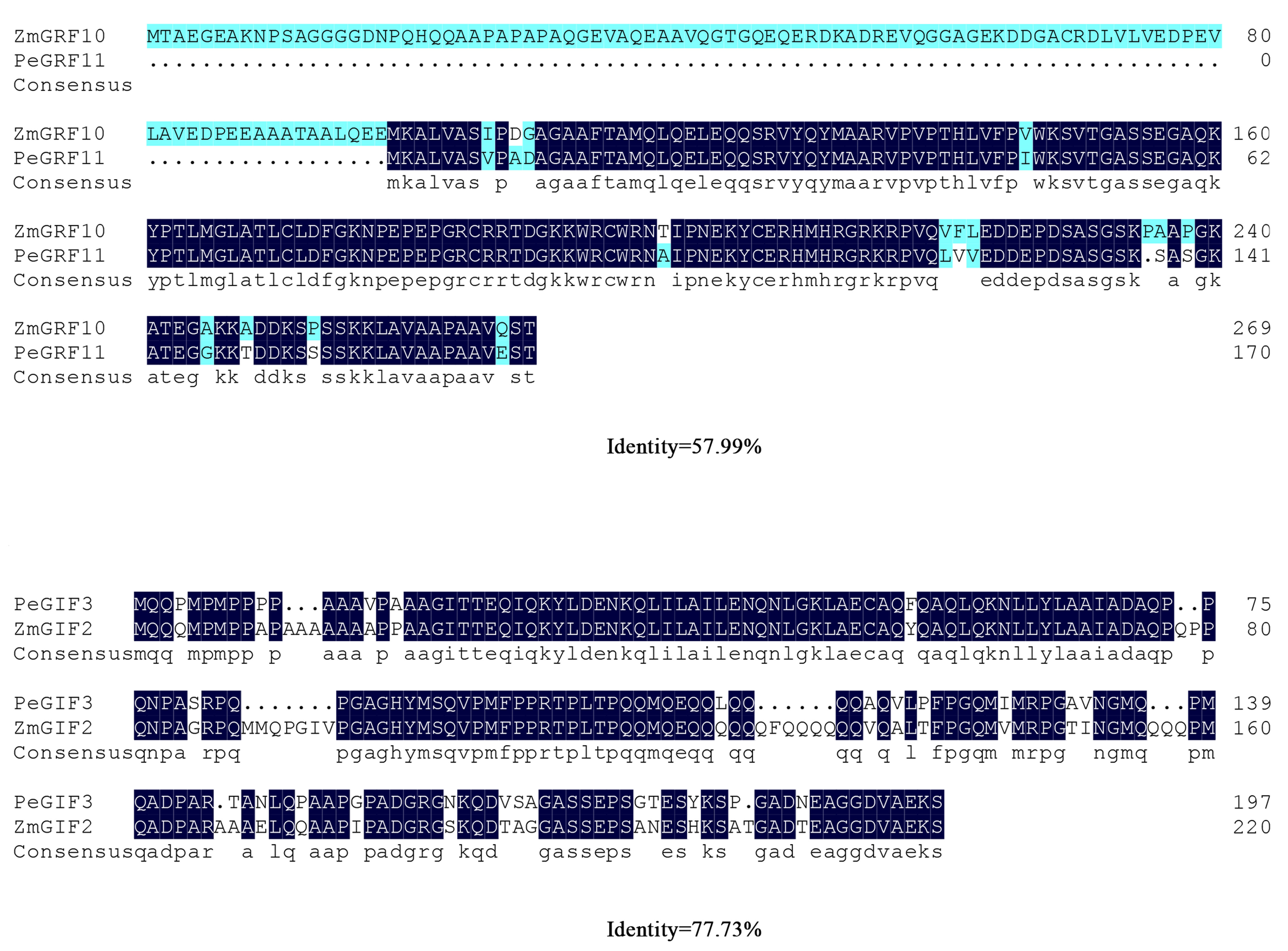

Supplement: Supplemental Information 14 [file peerj-07-7510-s014.png]
